# Structural basis for cross-reactivity and conformation fluctuation of the major beech pollen allergen Fag s 1

**DOI:** 10.1038/s41598-018-28358-1

**Published:** 2018-07-12

**Authors:** Adolfo H. Moraes, Claudia Asam, Fabio C. L. Almeida, Michael Wallner, Fatima Ferreira, Ana Paula Valente

**Affiliations:** 1 Chemistry Department, Federal University of Minas Gerais, Belo Horizonte, Brazil; 2 Department of Molecular Biology, University of Salzburg, Salzburg, Austria; 3 National NMR Center, Department of Structural Biology, Federal University of Rio de Janeiro, Rio de Janeiro, Brazil

## Abstract

Fag s 1 is a member of the Pathogen Related protein family 10 (PR-10) and can elicit cross-reaction with IgE antibodies produced against the birch pollen allergen Bet v 1. The Nuclear Magnetic Resonance (NMR) structure of Fag s 1 is presented along with its dynamic properties. It shares 66% identity with Bet v 1 and exhibits the expected three α-helices and seven β-sheets arranged as a semi-beta barrel and exposing the residues mapped as the Bet v 1 IgE epitope. The structural dynamics of Fag s 1 were monitored on the fast and intermediate timescales, using relaxation rates. The complex dynamics of Fag s 1 are closely related to the internal cavity, and they modulate IgE and ligand binding.

## Introduction

Fag s 1 is a member of the pathogenesis-related protein family 10 (PR-10), which are ubiquitously found in plants. These proteins are virtually absent in non-infected plants and are induced after pathogen infection or under other adverse conditions. The PR-10 proteins are unusual because they are intracellular, ubiquitous in nature, and some members can elicit allergic reactions in atopic individuals^1,2^. Fag s 1 can elicit cross-reaction with IgE antibodies produced against the birch pollen allergen Bet v 1. Birch pollen is one of the most common causes of rhinoconjunctivitis and allergic asthma in Northern and Central Europe and North America.

Individuals with birch pollen allergies can develop immediate reactions to fruits and vegetables in addition to seasonal respiratory symptoms. A birch pollen-related food allergy is considered a consequence of immunologic cross-reactivity between ubiquitous birch pollen allergens and structurally-related food proteins. IgE antibodies specific for the primary birch pollen allergen, Bet v 1, have been shown to cross-react with homologous proteins identified in various fruits, such as apple (Mal d 1), cherries (Pru av 1), and pears (Pyr c 1), as well as hazelnuts (Cor a 1), celery (Api g 1), carrots (Dau c 1), soybeans (Gly m 4), peanuts (Ara h 8), jackfruit, and kiwi (Act d 8)^3^. It is not clear which features are important in defining the allergenicity of PR-10 proteins, despite several structures having been elucidated either by Nuclear Magnetic Resonance (NMR) or X-ray crystallography. Among certain homologous allergens, little or no cross-reactivity has been observed. Therefore, the molecular definition of cross-reactivity clusters cannot rely solely on sequence homology; it requires experimental studies.

Members of the Bet v 1 family share their structural arrangements of β-α2-β6-α with an antiparallel β-sheet. The most striking feature of the Bet v 1 fold is the presence of an internal cavity that functions as a ligand-binding site and is therefore related to the biological function of these protein^4^.

Despite similarity in tertiary structures, members of the Bet v 1 family are very diverse in functionality. They serve as lipid binding and transfer proteins, mono or di-oxygenases, hydrolases, etc.^5–8^. *In vitro*, Bet v 1 can bind several hydrophobic compounds such as the plant hormone brassinosteroid, cytokinins, flavonoids, and fatty acids^6^. Quercetin-3-O-sophoroside was isolated bound to Bet v 1 from natural birch pollen and is proposed to be its physiological ligand^9^.

Recently, we proposed that structural dynamics are important for the allergenicity of Bet v 1^10^. Recombinant and naturally-occurring Bet v 1 were characterized structurally as well as immunologically in the presence and absence of the ligand, deoxycholate (DOC), and an animal model of allergic sensitization was also tested. Moreover, human IgE binding to Bet v 1 was analyzed using NMR spectroscopy. Binding of DOC stabilizes the IgE epitopes on Bet v 1 and the epitopes remain unaltered. Therefore, we speculate that humans are exposed to both ligand-bound and free Bet v 1 during sensitization and that the ligand-binding cavity of the allergen and related changes in structural dynamics are key structural elements for allergenicity^10^. Using relaxation rates, quantitative analysis performed by Grutsch *et al*. (2014) confirmed that ligand binding to the interior cavity leads to a compaction of the three-dimensional structure accompanied by a decrease in chemical exchange in the protein backbone and formation of a less dynamic conformer^11^.

Bet v 1 assumes various conformations in the free state that might represent the cavity opening, thus permitting the ligand to enter and bind to it. This conformational exchange process could also be important for other proteins with similar folds Therefore, characterizing the mechanisms of these transitions at the atomic level can significantly impact our understanding of the biological functions of many PR-10 proteins and shed light on the differences in allergenicity observed among isoforms, mutants, and related proteins.

In this work, we determined the NMR solution structure and the structural dynamics of Fag s 1 by monitoring on the fast and intermediate timescales. This work shows that the conformational exchange around the inner cavity of Fag s 1 is responsible for its complex dynamics.

## Results

### NMR solution structure of Fag s 1

**Figure 1:**
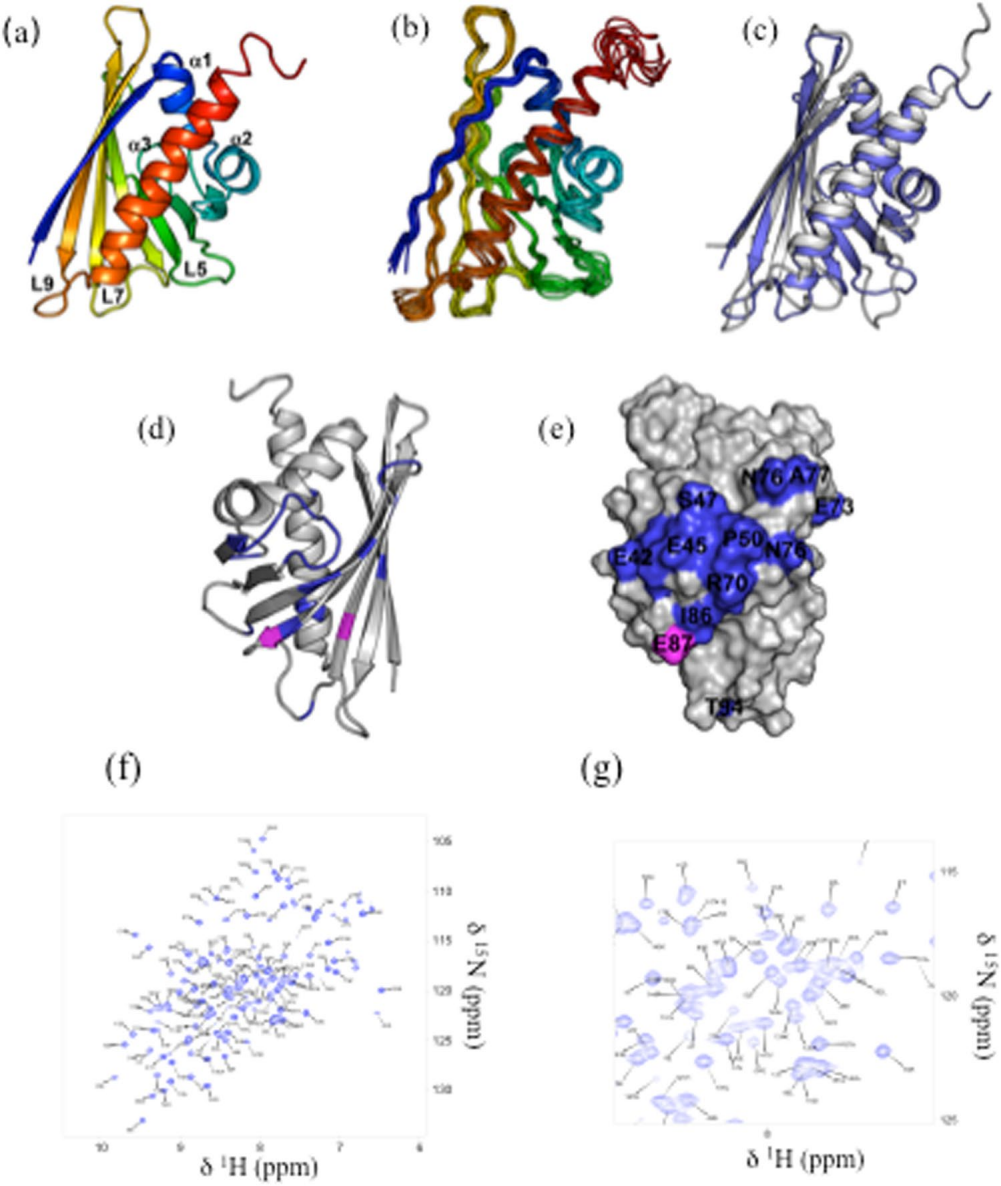
NMR Solution Structure of Fag s 1. (**a**) Lowest energy structure of Fag s 1 in cartoon representation; (**b**) Ensemble composed by the 10 lowest energy structure of Fag s 1 (6ALK); (**c**) Superposition of Fag s 1 NMR structure in blue (PDB) and Bet v 1 in grey (4A80); (**d**) and (**e**) Antibody epitopes identified for Bet v 1 on Fag s 1 structure: Antibody epitope of Bet v 1 identified by Mirza *et al*.^17^ and Asam *et al*.^10^ colored in magenta and blue, respectively; (**f**) ^1^H-^15^N HSQC spectrum showing the assignments and (**g**) zoom region.

The solution structure of Fag s 1 was calculated using NMR restraints, and it is composed of three α-helices and seven β-strands arranged as a curved semi-beta barrel. Figure 1a shows the lowest-energy structure as a ribbon representation, and Fig. 1b shows an ensemble of the ten lowest-energy structures. Three α-helices, α1, α2, and α3 are formed by residues P15–F22, A26–V33, and E130–E152, respectively. Residues V2–T11, I38–I43, I53–F58, Y66–D75, T80–G88, L95–A106, and S112–T122, are organized in β-strands β1-β7. The structure calculation statistics are summarized in Supplementary Table 1

Figure 1c shows the superposition of Fag s 1 (PDB ID: 6ALK) on Bet v 1 (PDB ID: 4A88)^4^. The two structures are similar, showing small differences in α1 and in the C-terminal of α3 (Fig. 1c). Figure 1d,e show the Fag s 1 structure in a ribbon representation and its surface, highlighting the antibody epitope mapped in Bet v 1 using NMR and X-ray. Most of the mapped epitope is conserved in the Fag s 1 surface except for N65, I71, N76, A77, T117 and K145. Supplemental Fig. 1 shows the primary sequence alignment of several Bet v 1 homologs including Fag s 1, demonstrating that the epitope is conserved and present in all the sequences.

Supplementary Table 2 summarizes the known structural information for Bet v 1 homologs compared with Fag s 1. All show strong similarities in their primary sequence (up to 71%) and tertiary structures. The Dali score between Fag s 1 and Bet v 1 is 19.3^2,3,12^ The cavity volume of Bet v 1 and its homologs is between 2,000 and 3,000 Å^3^ (Supplemental Table 2). None of those features has a direct correlation with hypersensibility.

### Backbone molecular dynamics of Fag s 1 by NMR spectroscopy

**Figure 2:**
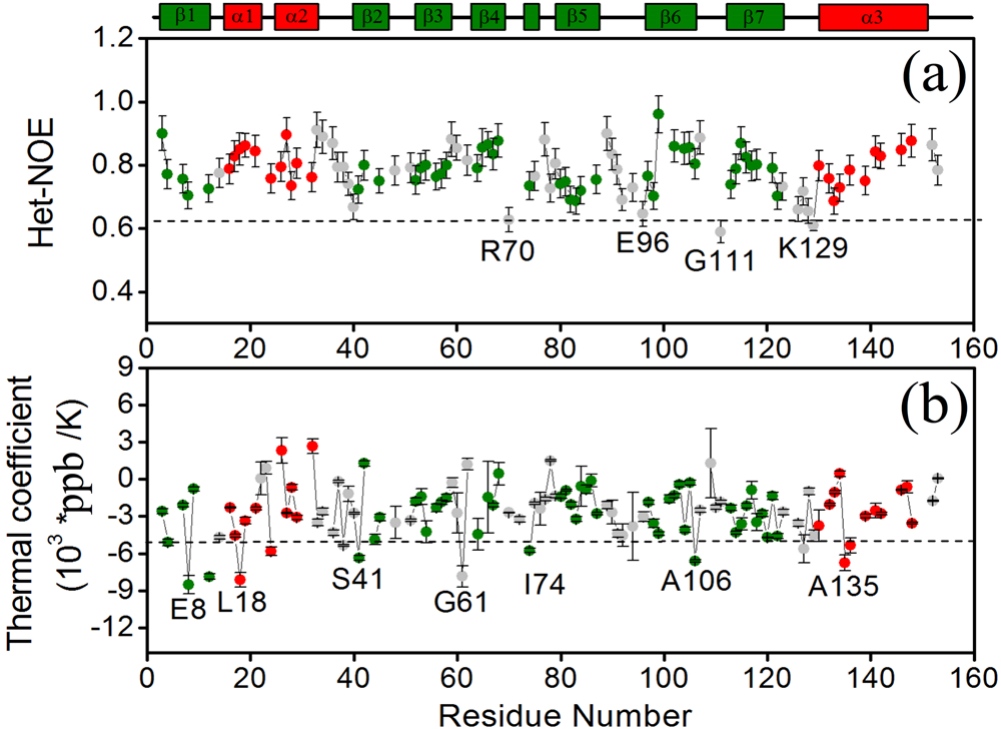
Structural dynamics of Fag s 1 at ps-ns timescale: (**a**) ^1^H-^15^N Heteronuclear NOE (Het-NOE) and (**a**) (TCI) Thermal coefficient index as a function of primary sequence residue number of Fag s 1.

The molecular dynamics of Fag s 1 in the ps-ns timescale were monitored by examining the relaxation rates (R_1_ and R_2_) and the ^1^H-^15^N Heteronuclear NOE (Het-NOE) (Figs S2a,b and 2a). It was found to be monomeric (τ_c_ 8.2 ns), rigid, having few residues – R70, E96, G111, I127 and K129 – with ^1^H-^15^N Het-NOE values below 0.65 (Fig. 2a).

The amide proton temperature coefficient has been used to predict hydrogen bond donors, and values more positive than −5 ppb/K are indicators of intramolecular hydrogen bonding^13^. The thermal coefficient index (TCI-ppb/K) of Fag s 1 was measured (Fig. 2b), and most of the NHs exhibited TCI values greater than −5 ppb/K, suggesting the presence of intramolecular hydrogen bonds, which it is consistent with the secondary structure elements except for E8, L18, S41, I74, A106, A135 and G136. The TCI values for the loop residues V12, L24, I38, G61, and E127 of Fag s 1 were less than −5 ppb/K. These results are consistent with a well-structured protein.

### Conformational fluctuation of Fag s 1 as determined by relaxation dispersion experiments

**Figure 3:**
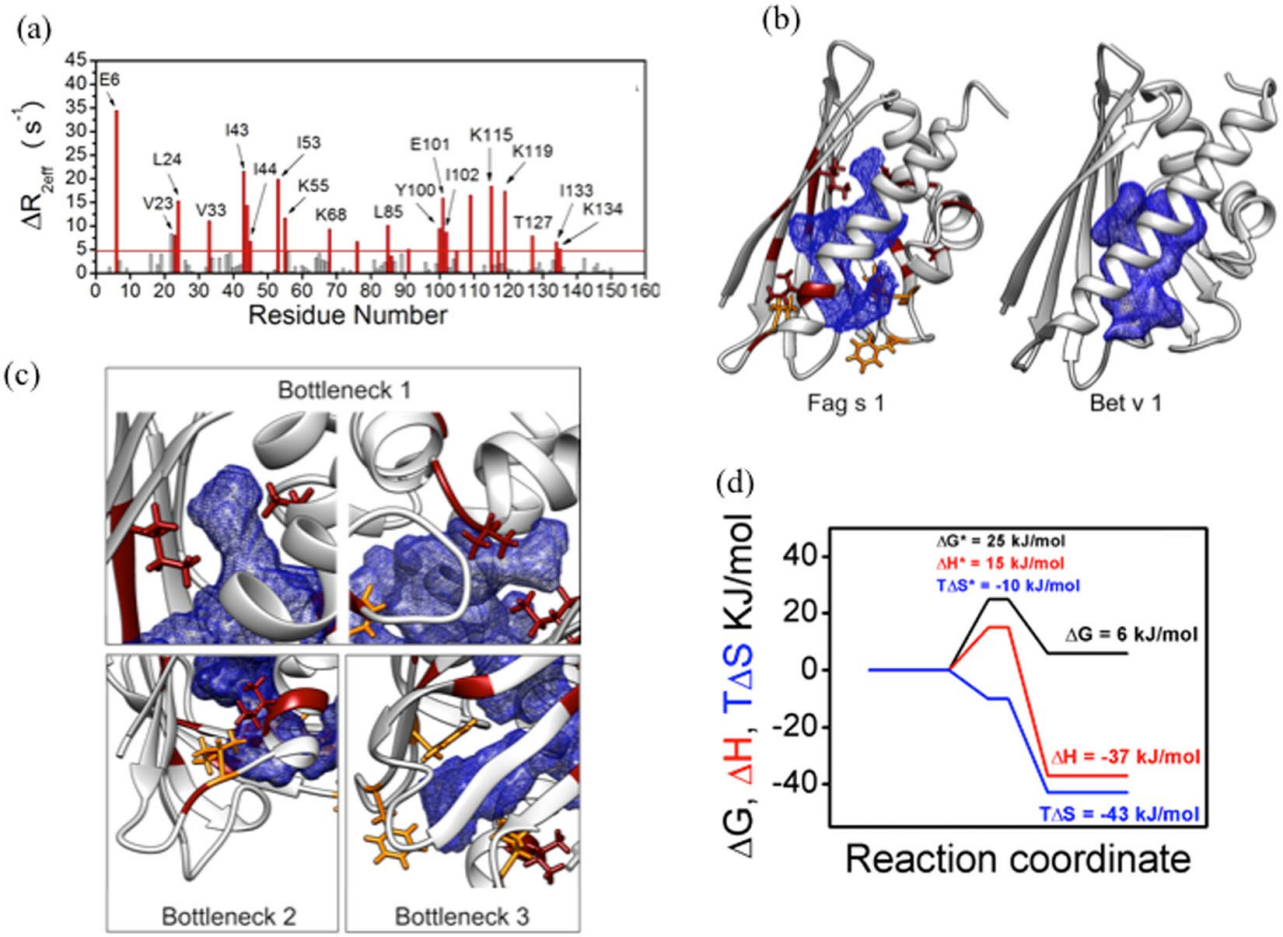
Conformation exchange in μs-ms timescale in Fag s 1 major cavity. (**a**) Difference between R_2eff_, ΔR_2eff_ obtained using the lowest and the highest CPMG frequency (66.7 and 1000 Hz) as a function of Fag s 1 residues number. Residues showing ΔR_2eff_ higher than 5 Hz are colored in red. (**b**) Fag s 1 and Bet v 1 cavity mapped using 3V as described in Material and Methods. Residues in conformation exchange are colored in red and the side chains that point towards the cavity are also shown. Residues with broadened resonances are colored in yellow (**c**) Zoom of some regions of Fag s 1 cavity showing crucial side chains forming specific bottlenecks; (**f**) Reaction coordinate diagram at 298 K.

The molecular dynamics of Fag s 1 in the μs-ms timescales were monitored experimentally by relaxation-compensated ^15^N-CPMG experiments (RC-^15^N-CPMG)^14^. Figure 3a shows the values obtained for *R*_*2,eff*_ *(s*^*−1*^) when ν_CPMG_ of 66.7 and 1000 Hz and the difference between them (*ΔR*_*2,eff*_ *(s*^*−1*^)*)* as a function of the residue number of Fag s 1. A difference greater than 5 Hz was used to identify residues undergoing conformational exchange in the fast-to-intermediate regime on the NMR chemical shift timescale. Supplementary Figure 3 and 4 show the relaxation dispersion curves for selected residues.

In Fag s 1 and Bet v 1 some residues were identified as undergoing conformational exchange and side chains were found to point toward the cavity (Fig. 3b). The relaxation dispersion profile of four residues (F22, F58, F64 and L128) could not be evaluated because they showed broadened NMR signals and small signal to noise ratio, an indication of exchange. Figure 3c shows a detailed view of the of Fag s 1 cavity. The side chains of residues in conformational exchange are oriented in “bottlenecks” in the cavity, suggesting a correlation between movements on the μs-ms timescale experienced by Fag s 1 residues and fluctuations in the cavity shape and volume. For instance, the hydrophobic side chains of residues F22 in α1, L23 in L2, and I102 in β6 form “bottleneck 1” of the Fag s 1 cavity. Two phenylalanines, F58 in β5, and F64 in L5, form “bottleneck 2,” and the side chains of residues I133 in the N-terminal of helix α3 and I128 in L9 form the nearby “bottleneck 3.”

Quantitative analysis of the conformational exchange of Fag s 1 was carried out by adjusting relaxation dispersion data using a theoretical global model of conformational exchange between two states: the ground state and the excited state. Relaxation dispersion curves were measured at 298, 300.5, 303, 305.5 and 308 K. From the theoretical treatment of relaxation dispersion curves acquired at five different temperatures, kinetic parameters could be obtained: exchange constant (k_ex_) between the ground state (A) and the high-energy conformation state (B); the populations of each state (p_A_ and p_B_), and their difference in the ^15^N chemical shift Δϖ_15N_. From these values, obtained at 298, 300.5, 303, 305.5, and 308 K (Supplementary Table 3), Van’t Hoff and Eyring plots were constructed, providing the energetic characterizations of high-energy state and transition states, respectively.

Data show that Fag s 1 residues are fluctuating at a *k*_ex_ rate of 812 ± 60 s^−1^ and p_b_ 8.5% at 298 K.

Kinetic and thermodynamic analyses of the conformational exchange process experienced by Fag s 1 were performed using the reaction coordinate diagram shown in Fig. 3d. This diagram was constructed using the Gibbs free energies, enthalpies and entropies of the major state, transition state and high-energy state, with ΔG_BA_ = 6 kJ/mol, at 298 K. The higher-energy conformation state is entropically unfavorable (TΔS = −43 kJ/mol) and enthalpically favorable (ΔH = −37 kJ/mol).

### Correlation between conformational exchange and interaction of Fag s 1 with dehydroergosterol (DHE)

Bet v 1 promiscuously binds molecules that include fatty acids, flavonoids, and cytokinins^5^ as well as its putative physiological ligand quercetin-3-O-sophoroside^8^. Using the approach of Mogensen *et al*. (2002), that is an Anilo-8-Naphthalene Sulfonate (ANS) displacement assay, we evaluated the binding capacity of Fag s 1 with known Bet v 1 ligands such as Deoxycholate (DOC), Sodium Dodecyl Sulfate (SDS), quercetin, and dihydroergosterol (DHE)^6^. Fag s 1 interacted with all tested compounds with dissociation constants in the micromolar range, similar to that found Bet v 1, with the exception of quercetin, which interacted with much lower affinity (Fig. S4).

**Figure 4:**
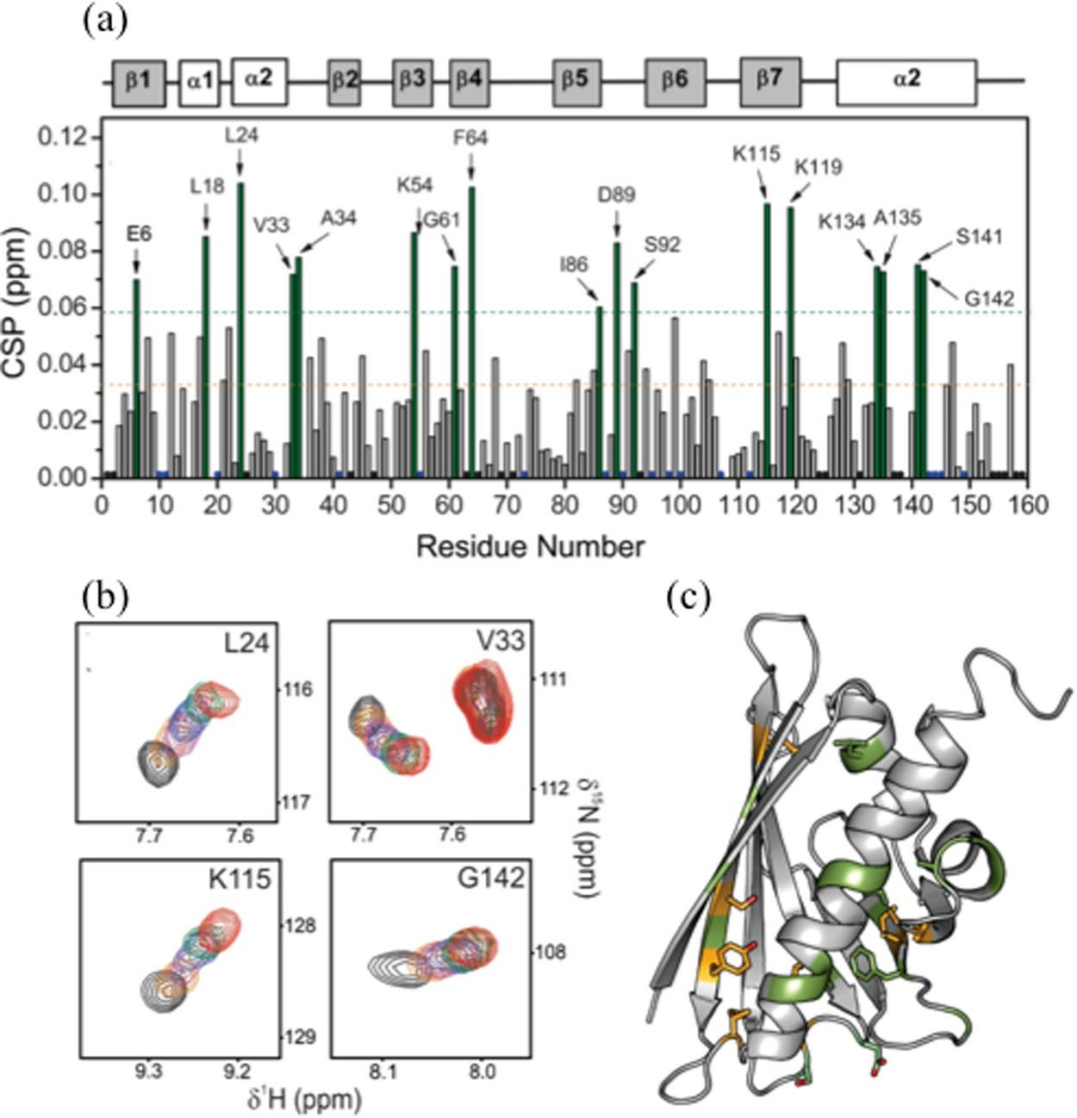
Chemical shift perturbation of Fag s 1 resonances upon titration with DHE. (**a**) CSP as a function of residue number at the highest titrated DHE concentration. Residues in green showed CSP higher than the mean plus one standard deviation. Black dots represent residues whose NH peaks does not appear in the HSQC even in the free state and blue peaks with signal overlaps (**b**) Spectra of selected residues in different DHE concentrations. (**c**) Residues mapped in (a) are colored green in the Fag s 1 structure and in yellow those that disappeared during titration (I38, I56, I91, L104, S118, Y120, and I127).

The binding of Fag s 1 with DHE was monitored using NMR spectroscopy in titration experiments. The chemical shift perturbation (CSP) index for the titration assay of Fag s 1 with DHE is shown in Fig. 4a. The NMR peaks of some residues at various concentrations of DHE are shown in Fig. 4b, and residues with higher CSP indices mapped onto the Fag s 1 solution structure (Fig. 4c). The NMR peaks of some residues disappeared during titration (in yellow in Fig. 4c).

**Figure 5:**
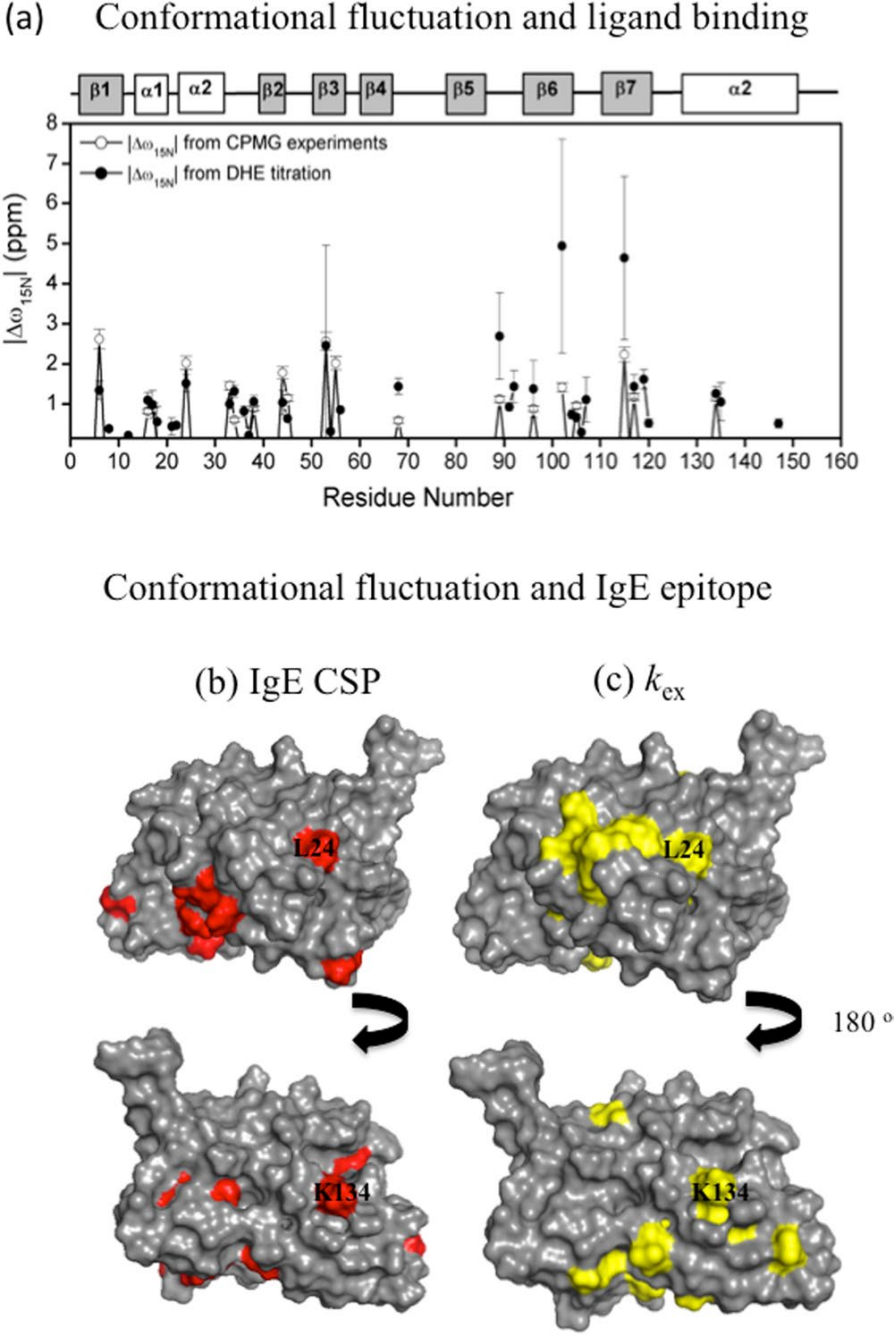
Correlation of conformational fluctuation and ligand binding and IgE epitope. (**a**) Comparison between Δω_15N_ obtained by DHE titration (black) and relaxation dispersion experiments (red). The results show similarity, except for residues 102 and 115 in β6 and β7, suggesting structural resemblance between the high-energy conformational state and the DHE bound state. (**b**) Residues in conformation exchange and with CSP in the presence of IgE^10^ and in conformational exchange (**c**) are mapped in the surface of Fag s 1 structure.

Several residues involved in DHE binding also underwent conformational exchange on the μs-ms timescale, as mapped by relaxation dispersion experiments. To better understand this behavior, ^15^N chemical shift variations (Δω_15N_), obtained by adjusting the DHE titration curves, were compared with those obtained by theoretical analysis of the relaxation dispersion experiments (Fig. 5a). It is worth noting that values obtained by titration and relaxation dispersion experiments were similar, except for residues 89 and 127 in β6 and β7. This result suggests structural similarity between the high-energy conformational state and the DHE-bound state.

## Discussion

As with other proteins in the PR-10 family, the Fag s 1 NMR solution structure is composed of seven β-strands and three α-helices forming a secondary structure arrangement (β-α2-β6-α) with an antiparallel curved β-sheet. Those secondary structures fold into one tridimensional structure in which the main characteristic is the presence of a large hydrophobic or amphipathic cavity. This cavity plays a pivotal role in the function of all PR-10 proteins in ligand and IgE binding^8–12,15–17^.

To illustrate this, we aligned Fag s 1 with Bet v 1 and eight other allergenic PR-10 proteins whose structures have already been determined, focusing on the Bet v 1-antibody epitopes. Mirza *et al*. solved the structure of the Bet v 1-Fab BV16 complex and from this structure it was possible to identify one conformational epitope composed of residues E42-T52 in the PR-10 highly-conserved p-loop, R70 and D72 in β4, H76 in loop L4, and K97 in β6^17^. Another important epitope-identifying study was carried out by Asam *et al*. (2014), who used polyclonal antibodies from Bet v 1-sensitive patients to identify conformational epitopes of Bet v 1 by NMR spectroscopy^10^. The location and composition of the Bet v 1-identified epitopes on Fag s 1 and Bet v 1 structures are conserved, except for residues N65, I73, A76, T117 and K145 (Fig. S1). The primary consequence of the great similarity between Fag s 1 and Bet v 1 is the cross-recognition of Fag s 1 by antibodies induced by Bet v 1 sensitization.

In contrast to its rigidity on the ps-ns time-scale, the Bet v 1structure is very dynamic at the μs-ms time-scale^10,11^. Movements at this time-scale are related to chemical conformational exchange, which contributes to the structural malleability of proteins, an essential characteristic for their biological functions. Usually, in order to perform their functions, proteins exist in different conformational states that can be representing intermediates of a folding process, mimicking of a bound state, or intermediates in a catalytic pathway^18–21^. Uniquely, NMR spectroscopy is a technique that allows characterization of μs-ms timescale movements in proteins and, consequently, provides important information about higher-energy conformational states^22–24^.

The molecular dynamics of Fag s 1 and its conformational equilibrium were characterized by relaxation-compensated ^15^N relaxation dispersion experiments^14,20,25^. Many residues of Fag s 1 showed conformational exchange contributions in their *R*_*2,eff*_ *(s*^*−1*^*)* rates revealing that Fag s 1 assumes a ground state and a high-energy state in the apo form, as evidenced by the conformational dynamics on a μs-ms timescale.

Many of the identified residues have side chains located in the Fag s 1 cavity. Because many residues showing conformation exchange in the relaxation experiments were “strategically” positioned in bottlenecks in the Fag s 1 cavity, the idea of correlation between atomic movements on the μs-ms timescale as detected by NMR and fluctuations in the cavity shape and /or volume was reinforced.

Fluctuations in the internal cavity volume and/or shape seem to be the structural elements responsible for conformational variability. The “opening” and “closing” of the cavity could be responsible for formation of the higher energy state. Such transient conformational rearrangements could be necessary to generate a channel through which the ligand can enter the cavity, and they would include movement of the backbone, rearrangement of the loops and movement of the side chains.

Internal cavities in proteins are crucial structural elements that influence their dynamics and stabilities. For example, Phage T4 lysozyme is a well-studied protein, and its mutant, L99A, containing a large internal cavity capable of binding hydrophobic molecules, is one of the best-characterized systems. The creation of the cavity did not involve a change in structure but is related to protein dynamics, mainly involving residues close to the cavity^26^. In this case, the greatest difference between the ground and excited states is the F114 side chain, which is solvent-exposed in the major state and occupies the cavity created by the L99A mutation, becoming buried in the core.

Proteins face major challenges during the binding of hydrophobic molecules. They need to expose hydrophobic regions to permit entrance by the ligand, but as a result they can become vulnerable due to sampling of conformational states that present a risk of misfolding and aggregation^26–28^. Evolutionary pressure creates competition between increased binding efficiency and reduced protein stability, balancing the ability to breathe and populate multiple states without creating an unacceptable propensity for aggregation^29^.

Furthermore, conformation fluctuation close to the epitope-antibody binding site can modulate IgE affinity. We attempted to correlate the residues that exhibit conformation fluctuation with those that exhibit CSP in the presence of IgE, as reported by Asam *et al*. Fig. 5b,c show that these residues populate similar regions. The presence of residues in conformational exchange close to the binding site is a very common feature that is important for modulating affinity. Recently Yanaka *et al*. (2017), proposed a method called fluctuation editing, which identifies potential mutation sites using relaxation dispersion experiments^30^. Using this strategy, they were able to engineer a higher affinity antibody. Our data show potential sites for mutation that can help engineer a different allergen with special IgE binding properties.

## Materials and Methods

### Expression and Purification of Isotope-labeled (^15^N, ^13^C) Fag s 1

The sequence of Fag s 1 1.0101, termed Fag s 1 in the following, was inserted into a pET28b (Novagen, Merck KGaA, Darmstadt, Germany) vector and transformed and expressed in *Escherichia coli* BL21 Star™ (DE3) cells (Invitrogen, Carlsbad, CA, USA). Expression of recombinant ^15^N- and ^13^C^15^N-isotope-labeled protein was performed in *E. coli* OD 2 N and *E. coli* OD 2 CN medium, respectively, (Silantes, München, Germany) supplemented with 25 mg/L kanamycin. After induction of protein expression with 0.5 mM isopropyl-β-D-thiogalactopyranoside (IPTG) at an OD600 nm of 0.8, growth of the cells was continued for 20 h at 16 °C. Cells were harvested by centrifugation and cell pellets dissolved in 1/50 culture volume of 20 mM sodium phosphate buffer pH 8.0. Cell breakage was performed using liquid nitrogen. To supernatants collected after centrifugation at 15,000 g, 0.5 M sodium chloride and 0.15 M NaH_2_PO_4_ were slowly added while stirring on ice for 30 minutes. After a further centrifugation step at 15,000 g the supernatant was filtered through a 0.45 µm filter and applied to a 5-mL Phenylsepharose column (GE Healthcare Biosciences, Little Chalfont, UK). The protein was eluted with 25 mM Tris/HCl pH 9.3, 8% (v/v) 2-propanol and a final purification was performed by size exclusion chromatography in 10 mM sodium phosphate buffer pH 8 using a Superdex 75 10/300 GL column (GE Healthcare Biosciences). The recombinant protein was lyophilized and stored at −20 °C.

### NMR Spectroscopy

The NMR experiments were performed at 308 K using Bruker Avance III 800 and 700 spectrometers equipped with TXI 5 mm triple-resonance probe and a Bruker Avance III 600 equipped with TCI 5 mm triple resonance cryogenic-probe. The assignment was done following procedures previously published by Moraes *et al*.^31^. For the assignment of the NMR resonances of the backbone atoms the following NMR triple-resonance experiment were analyzed: ^1^H-^15^N HSQC, ^1^H-^13^C HSQC, HNCO, HN(CA)CO, HNCA, HNCACB and CBCA(CO)NH. Side-chain assignments were done through the analysis of HBHA(CBCA)(CO)NH. HCCH-TOCSY, HCCH-COSY. The assignment was completed and confirmed using ^15^N-edited NOESY-HSQC (100 ms mixing time) ^13^C-edited NOESY-HSQC (100 ms mixing time). The spectra were processed using Topspin 2.1 (Bruker Corporation, USA) and analyzed using CCPN^32^.

### Structure calculation

The solution structure calculation of Fag s 1 was carried out using experimental restraints obtained by different solution NMR experiments. Distance restraints were obtained from 3D-^13^C-edited and ^15^N-edited NOESY-HSQCs experiments. Dihedral angles restraints were achieved from backbone chemical shifts processed with the software Talos-N^33^. The NOE assignments were carried out in a semi-automatic way using the software Aria 2.3^34^. The structure calculation was performed with the software CNS Solve 1.2^35^, using simulating annealing protocol and torsion molecular dynamics. The 20 lowest energy structures were selected for representing the Fag s 1 solution structure from 400 calculated at step 8 of the interactive assignment and structure calculation of Aria. Actually, the protocol uses 30 starting structures in each step up to step 7. The quality of the obtained ensemble was verified using “Protein Structure Validation Suite”, PSVS (http://psvs-1_5-dev.nesg.org/).

### Cavity volume calculation and identification

Cavity-volumes of Fag s 1, Bet v 1 and the other PR-10 proteins listed in Table S1 were calculated using software Mole 2.0^36^ and the webserver 3V^37^. The algorithm of Mole 2.0 is based on the computation of the Delaunay triangulation/Voronoi diagram of the atomic centers and further construction of the molecular surface, and identification of cavities. Cavities identified by Mole 2.0 should be analyzed carefully since they are geometric entities used to tunnel and pore identification. Differently, 3V webserver uses what is known as rolling-probe strategy, where the cavity is identified by taking the difference between the volume obtained by rolling on small probe sized similarly with the solvent radios and another probe with radius as large as possible. Both softwares have as refine parameters the size of both probes. In this work, we standardized smaller probe and bigger probe with radius of 1.3 Å and 3.0 Å, respectively.

### Backbone dynamics

The backbone molecular dynamics of Fag s 1 were characterized using the relaxation rates R_1_, R_2_ and {^1^H-^15^N}-Heteronuclear NOE (Het-NOE) obtained from ^15^N relaxation experiments acquired in Bruker Avance III 500 MHz spectrometer. R_1_ and R_2_ rates were obtained from the fitting as a single intensity exponential decay of resonance intensities as a function of the delays: 0.05, 0.1, 0.2, 0.3, 0.5, 0.6, 0.8, 0.9 and 1.0 s for R_1_ and 16.96, 33.92, 50.88, 67.84, 84.8, 101.76, 118.72, 135.68, 203.52 and 237.44 ms for R_2_, respectively^38,39^. {^1^H-^15^N}-Heteronuclear NOE rates were acquired from ^1^H-^15^N correlation experiments with and without saturation of ^15^N magnetization, using recovery time of 5.0 s^39^.

### Fag s 1 molecular dynamics on μs-ms timescale

The molecular dynamics of Fag s 1 on μs-ms timescale was assessed with relaxation-compensated ^15^N single quantum Carr-Purcell-Meiboom-Gill (CPMG) relaxation dispersion experiments (Loria *et al*. 1999). CPMG experiments were acquired in Bruker Avance III spectrometers with ^1^H Larmor frequency of 500 and 700 MHz at five temperatures: 298.0, 300.5, 303.0, 305.5 and 308.0 K. Experimental R_2_ effective rates (${R}_{2,\,eff}^{exp}$) were obtained from the intensities measured using the software CCPN analysis^32^ from ^1^H-^15^N correlation spectra and applying the following formula:

1 $${R}_{2,eff}^{exp}=-\frac{1}{{T}_{relax}}ln(\frac{{I}_{0}}{{I}_{CPMG}}),$$

where *T*_*relax*_ is the relaxation delay of 30 ms, *I*_*CPMG*_ is the intensity measured in ^1^H-^15^N spectra acquired with a CPMG frequency, ν_CPMG_, ranging from 66.7 to 1000 Hz. *I*_*0*_ is the peak intensity obtained with ${T}_{relax}$ of 0 ms. The relaxation dispersion CPMG experiments were acquired with the following ν_cpmg_ 66.7, 133.3, 200, 266.7, 333.3, 400, 466.7, 533,3, 600, 666.7, 866,7 and 1000 Hz. Experimental errors of *R*_*2eff*_ *(s*^*−1*^) were calculated using the following equation:

2 $${\rm{\Delta }}{R}_{2,eff}^{exp}=-\frac{\sigma }{T{I}_{CPMG}},$$

where σ and *I*_*CPMG*_ are the standard deviations of noise, and NMR resonance intensity measured from spectrum acquired with a *ν*_*CPMG*_ of 500 Hz. A minimum error of 3% of the *R*_*2eff*_ *(s*^*−1*^) values where used when the experimental errors calculated using equation 2 where lower than this imposed value.

The experimental *R*_*2,eff*_ *(s*^*−1*^) values as a function of ν_cpmg_ were analyzed adjusting a theoretical model built up applying the numerical solution of Bloch-McConnell equations to a system in equilibrium between two states: a high-populated A state, ground state, and a low-populated B state, excited state^40^. The software CPMG_fit (Korzhnev and Kay, 2008) was used for fitting the dispersion curves and for extraction of kinetic and thermodynamic parameters. In this step, residues presenting overlaps on the ^1^H-^15^N HSQC spectra, those showing experimental results with low quality, and those in which the difference on *R*_*2eff*_ *(s*^*−1*^) obtained with ν_CPMG_ of 66.7 (lowest value) and 1000 (highest value) was smaller than 5 Hz were excluded.

At the end, ^15^N-RC-CPMG relaxation rates of *n*_*R*_ = 22 amide groups, acquired at *n*_*T*_ = 5 different temperatures and at *n*_*B*_ = 2 magnetic fields were least-square fitted together by minimizing the following target function:

3 $${\chi }^{2}(\zeta )={\sum }^{}{(\frac{{R}_{2,eff}^{calc}(\zeta )-{R}_{2,eff}^{exp}}{{\rm{\Delta }}{R}_{2,eff}^{exp}})}^{2},$$

where ${R}_{2,\,eff}^{calc}(\zeta )$ are model relaxation rates, ζ = {χ_1_, …, χ_npar_} is the set of adjustable parameters, n_par_ is the number of adjustable parameters, ${R}_{2,\,eff}^{exp}\,\,$are the experimental relaxation rates, and ${\rm{\Delta }}{R}_{2,\,eff}^{exp}$ are the experimental uncertainty associated with them. The summation in equation (3) was done over the number of experimental points ndat = {n_R_, n_T_, n_B_}. The data fitting was carried out taking the following assumption: (a) all residues in Fag s 1 are involved in the same global exchange process (same equilibrium rate constant, *K*_*ex*_), (b) the intrinsic relaxation rates R_2,0_ are the same in states A and B, (c) the chemical shift differences between states for each residue, Δ*ϖ*_*BA*_, are independent of temperature, and (iv) CPMG experimental data were analyzed independently for each temperature. The following adjustable parameters were obtained: population of state B, *p*_*B*_, and, consequently, the equilibrium rate constant constant, *K*_*e*_ = *p*_*B*_*/p*_*A*_, rate constant *k*_*AB*_, n_R_^15^N chemical shift difference between states A and B, Δ*ϖ*_*BA*,_ and n_R_*n_B_ intrinsic relaxation rates, *R*_*2,0*_.

The Gibbs free energy difference of the states A and B, Δ*G*_*BA*_ = Δ*H*_*BA*_ – TΔ*S*_*BA*_, was obtained fitting the temperature dependence of the equilibrium constant, *K*_*ex*_, using the Vant’off equation:

4 $$\mathrm{ln}({K}_{e})=-\frac{{\rm{\Delta }}{G}_{BA}}{R}\frac{1}{T}=\frac{{\rm{\Delta }}{S}_{BA}}{R}-\frac{{\rm{\Delta }}{H}_{BA}}{R}\frac{1}{T},$$

where *R* = *8.314* *J* *K*^*−1*^ *mol*^*−1*^ is the gas constant, ${\rm{\Delta }}{S}_{BA}$ and ${\rm{\Delta }}{H}_{BA}$ are the entropy and enthalpy difference between states A and B, respectively, and T is the experimental temperature in K.

The transition state was analyzed using Transition State Theory. The temperature dependence of rate constant *k*_*AB*_ was fitted with the Eyring equation.

5 $$\mathrm{ln}(\frac{{k}_{AB}}{T})-\,\mathrm{ln}(\frac{\kappa {k}_{B}}{h})=-\frac{{\rm{\Delta }}{G}^{\ast }}{R}\frac{1}{T}=\frac{{\rm{\Delta }}{S}^{\ast }}{R}-\frac{{\rm{\Delta }}{H}^{\ast }}{R}\frac{1}{T},$$

where $\kappa ={1}{.}{{6}}^{\cdot 1}\,{1}{{0}}^{-7}$ is the transmission coefficient that is related to the fraction of molecules in the transition state that end up as products^41,42^, *k*_*B*_ and *h* are the Boltzmann and Planck constants, respectively. ΔG*, ΔH* and ΔS* are activation Gibbs free energy, activation enthalpy and activation entropy, respectively.

### Interaction of Fag s 1 with ligands

The interaction of Fag s 1 with quercetin, naringenin, kinetin, sodium dodecyl sulfate (SDS) (Sigma Aldrich, USA) was monitored by competitive binding assays with the fluorescent probe 1-Anilo-8-Naphthalene Sulfonate (ANS) (Sigma Aldrich, USA). Fluorescence spectra of 15 μM of ANS in solutions with different concentrations of Fag s 1 (0.5 to 50 μM) in 20 mM of saline phosphate buffer, 50 mM NaCl, pH 7.8 were acquired in Spectrometer Agilent Cary 100 (Agilent S. A, USA) equipped with temperature control at 25 °C. Competitive ligand-binding assays were done adding different concentrations of small compounds, listed above, to the solution of 10 μM of Fag s 1 and 10 μM of ANS. Except for SDS, all ligand-candidate compounds were previously solubilized in DMSO. The relative volume of DMSO in the final solution never exceeded 5% and the effect of DMSO on the fluorescence of Fag s 1-bound ANS was carefully verified.

The interaction of Fag s 1 with dehydroergosterol (DHE) (Sigma Aldrich, USA) was monitored by the chemical shift perturbation on the ^1^H-^15^N HSQC spectra upon ligand titration. In those experiments, the concentration of Fag s 1, ${[P]}_{0}$, was kept 80 μM, and the DHE concentration,$\,{[L]}_{0}$, ranged from 5 to 500 μM. The $|{\rm{\Delta }}{\omega }_{15N}|$ values between the free state and the DHE-bound state of Fag s 1 were calculated fitting the ^15^N chemical deviations upon titration, ${|{\rm{\Delta }}{\omega }_{15N}|}_{obs}$, as a function of DHE concentration,$\,{[L]}_{0}$, using the following equation^43^:

6 $$\,{|{\rm{\Delta }}{\omega }_{15N}|}_{obs}={|{\rm{\Delta }}{\omega }_{15N}|}_{max}\frac{({K}_{D}+{[L]}_{0}+{[P]}_{0})-\sqrt{{({K}_{D}+{[L]}_{0}+{[P]}_{0})}^{2}-4({[P]}_{0}{[L]}_{0})}}{2{[P]}_{0}},$$

where ${K}_{D}$ is the dissociation constant and ${|{\rm{\Delta }}{\omega }_{15N}|}_{max}$ is the chemical shift difference between the free and the DHE-bound state of Fag s, here and in the following sections of this work called $|{\rm{\Delta }}{\omega }_{15N}|$, for simplicity.

The experiments were acquired in a spectrometer Bruker 600 MHz Avance III equipped with TCI 5 mm triple resonance cryogenic-probe at 308 K. Changes in dynamics of Fag s 1 caused by DHE binding either in μs-ms were monitored by the same experiments used to track the molecular dynamics of free Fag s 1. Following these ideas our work contributes to the understanding of the correlation between dynamics, ligand binding, allergenicity and IgE recognition of PR-10 proteins.

### Accession numbers

Coordinates for the structure of Fag s 1 have been deposited in the protein Data Bank (PDB) with ID code 6ALK.

## Electronic supplementary material

Supplementary Information
