# Supplementary material for: Structural basis for cross-reactivity and conformation fluctuation of the major beech pollen allergen Fag s 1
Source: Sci Rep. 2018 Jul 12;8:10512. doi: 10.1038/s41598-018-28358-1 (PMC6043577; doi:10.1038/s41598-018-28358-1)
Supplement: Supplementary file 1 — Supplementary Information [file 41598_2018_28358_MOESM1_ESM.docx]

**Supplementary data**

**Structural basis for cross-reactivity and conformation fluctuation of the major beech pollen allergen Fag s 1**

Adolfo H. Moraes^1^; Claudia Asam^2^; Fabio C. L. Almeida^3^; Michael Wallner^2^; Fatima Ferreira^2^; and Ana Paula Valente^3*^

1- Chemistry Department, Federal University of Minas Gerais, Belo Horizonte, Brazil; 2- Department of Molecular Biology, University of Salzburg, Salzburg, Austria 3- National NMR Center, Department of Structural Biology, Federal University of Rio de Janeiro, Rio de Janeiro, Brazil.

**Supplementary Table 1: Lists of Restrains and Statistical Analysis for NMR Solution Structure of Fag s 1.**

| **Number of experimental restrains** |  |
| --- | --- |
| Total NOE distance restrains | 1966 |
| Ambiguous | 179 |
| Intra-residue | 643 |
| Sequential | 474 |
| Short range (2 ≤ \|i – j\| ≤ 3) | 171 |
| Medium range (3 < \|i – j\| ≤ 5) | 77 |
| Long range ( \|i – j\| > 5) | 422 |
| Torsion angle (phi/psi) | 177 |
| H-bonds restrains | 40 |
| **RMSD from average structure (Å)** |  |
| Backbone (3-23, 26-45, 49-58,66-87, 94-109, 112-122, 130-152, 154-156) | 0.7 |
| Backbone, all-residues | 1.0 |
| Heavy atoms (3-23, 26-45, 49-58,66-87, 94-109, 112-122, 130-152, 154-156) | 1.1 |
| Heavy atoms, all residues | 1.5 |
| **Restrains violations** |  |
| Noe violations (> 0.5 Å) | 3 |
| Dihedral violations (> 5°) | 0 |
| **Deviations from ideal geometry** |  |
| RMS for bond lengths (Å) | 0.005 |
| RMS for bond angles (°) | 0.7 |
| **Ramachandran plot of ordered residues (%)^b^** |  |
| Most favored regions | 80.8 |
| Allowed regions | 19.2 |
| Generously allowed regions | 0.0 |
| Disallowed | 0.0 |

^a^ Deviation from ideal geometry calculated using CNS Solve protocols.

^b^ The RMSD and calculation and PROCHECK analyses were performed for the ensemble of the 20 lowest energy structures using PSVS suite.

**Supplementary Table 2: PR-10 proteins in PDB in comparison with Fag s 1**

| Protein name | Source | Frequency of food hypersensibility (%) (a) | PDB code | Sequence ID | Structural Similarity with Fag s 1 (Dali) | RMSD | Cavity Volume  Å^3^ | Reference |
| --- | --- | --- | --- | --- | --- | --- | --- | --- |
| **Fag s 1** | Beech |  |  |  |  |  | 2,243 |  |
| **Bet v 1** | Birch |  | 4A88 | 64 | 19.3 | 2.5 | 2,337 | b |
| **Mal d 1** | Apple | 80 | 5MMU | 61 | 17.9 | 2.3 | 2,230 | c |
| **Dau c 1** | Carrot | 34.5 | 2WQL | 36 | 17.2 | 2.7 | 2,483 | d |
| **Pru av 1** | Cherry | 32.1 | 1E09 | 71 | 17.1 | 2.8 | 2,932 | e |
| **Ara h 8** | Peanut | 24.2 | 4W9B | 50 | 16.7 | 3.0 | - | - |
| **Api g 1** | Celery | 15.8 | 2BK0 | 41 | 17.3 | 2.6 | 2,846 | f |
| **Gly m 4** | Soya | 13.9 | 2K&H | 50 | 14.9 | 3.3 | 2,970 | g |
| **Hyp 1** | St. John’s wort | - | 3IE5 | 45 | 18.2 | 2.6 | 2,040 | h |
| **Fra a 1** | Strawberry | - | 2LPX | 64 | 14.6 | 3.2 | 3,649 | i |

a- Geroldinger-Simic M, Zelniker T, Aberer W, Ebner C, Egger C, Greiderer A, Prem N, Lidholm J, Ballmer-Weber BK, Vieths S, and Bohle B (2011) J. Allergy Clin Immunol 127(3): 616-622.

b- Kofler S, Asam C, Eckhard U, Wallner M, Ferreira F & Brandstetter H (2012) Crystallographically mapped ligand binding differs in high and low IgE binding isoforms of birch pollen allergen bet v 1. J Mol Biol 422, 109**–**123.

c- Ahammer L., Grutsch S, Kamenik AS, Liedl KR,and Tollinger M (2017) Structure of the Major Apple Allergen Mal d 1 J. Agric. Food Chem., 65, 1606**−**1612.

d- Markovic-Housley Z, Basle A, Padavattan S, Maderegger B, Schirmer T & Hoffmann-Sommergruber K (2009) Structure of the major carrot allergen Dau c 1. Acta Crystallogr D Biol Crystallogr 65, 1206**–**1212.

e- Neudecker P, Schweimer K, Nerkamp J, Scheurer S, Vieths S, Sticht H & Rosch P (2001) Allergic cross-reactivity made visible: solution structure of the major cherry allergen Pru av 1. J Biol Chem 276, 22756**–**22763.

f- Schirmer T, Hoffimann-Sommergrube K, Susani M, Breiteneder H & Markovic-Housley Z (2005) Crystal structure of the major celery allergen Api g 1: molecular analysis of cross-reactivity. J Mol Biol 351, 1101**–**1109.

g- Berkner H, Neudecker P, Mittag D, Ballmer-Weber BK, Schweimer K, Vieths S & Rosch P (2009) Crossreactivity of pollen and food allergens: soybean Gly m 4 is a member of the Bet v 1 superfamily and closely resembles yellow lupine proteins. Biosci Rep 29, 183**–**192.

h - Michalska K, Fernandes H, Sikorski M & Jaskolski M (2010) Crystal structure of Hyp-1, a St. John’s wort protein implicated in the biosynthesis of hypericin. J Struct Biol 169, 161**–**171.

i-Seutter von Loetzen C, Schweimer K, Schwab W, Rosch P & Hartl-Spiegelhauer O (2012) Solution structure of the strawberry allergen Fra a 1. Biosci Rep 32, 567**–**575.

**Supplementary Table 3: Statistics and values of kinetic and thermodynamic parameters obtained by global fitting of ^15^N RC CPMG relaxation dispersion data of Fag s 1.**

| **Temperature (K)** | **F** | **N. residues** | **DF^b^** | **k_ex_ (s^-1^)** | **p_B_ (%)** |
| --- | --- | --- | --- | --- | --- |
| **298** | 3204 | 22 | 2409 | 812 ± 60 | 8.5 ± 1.2 |
| **300.5** |  |  |  | 968 ± 92 | 7.1 ± 1.1 |
| **303** |  |  |  | 1090 ± 72 | 6.5 ± 1.0 |
| **305.5** |  |  |  | 1167 ± 95 | 6.0 ± 0.9 |
| **308** |  |  |  | 1630 ± 95 | 5.3 ± 0.8 |

FAG S 1 1 -GV-------FTYESETTTVITPARLFKAFVLDADNLIPKVAPQA**I**KSSEIIEGSGGPGT
BET V 1 1 -GV-------FNYETETTSVIPAARLFKAFILDGDNLFPKVAPQA**I**SSVENIEGNGGPGT
MAL D 1 1 -GV-------YTFENEFTSEIPPSRLFKAFVLDADNLIPKIAPQA**I**KQAEILEGNGGPGT
DAU C 1 1 MGA-------QSHSLEITSSVSAEKIFSGIVLDVDTVIPKAATGA**Y**KSVE-VKGDGGAGT
PRU AV1 1 -GV-------FTYESEFTSEIPPPRLFKAFVLDADNLVPKIAPQA**I**KHSEILEGDGGPGT
ARA H 8 1 MGV-------FTFEDEITSTVPPAKLYNAM-KDADSITPKII-DD**V**KSVEIVEGNGGPGT
API G 1 1 MGV-------QTHVLELTSSVSAEKIFQGFVIDVDTVLPKAAPGA**Y**KSVE-IKGDGGPGT
GLY M 1 1 -GV-------FTFEDEINSPVAPATLYKALVTDADNVIPKAL-DS**F**KSVENVEGNGGPGT
HYP 1 1 -GIDPFTMAAYTIVKEEESPIAPHRLFKALVLERHQVLVKAQPHV**F**KSGEIIEGDGGVGT
FRA A 8 1 MGV-------YTYENEFTSDIPAPKLFKAFVLDADNLIPKIAPQA**V**KCAEILEGDGGPGT
consensus 1 *. .... * .. ... ...............*.........*...*.**.**

FAG S 1 53 IKKITFGEGSQF**N**YVKH**RI**D**E**ID**NA**NFTYACTL**I**EGDAISE**T**LEKIAYEI**K**LVASPDGGS
BET V 1 53 IKKISFPEGFPF**K**YVKD**RV**D**E**VD**HT**NFKYNYSV**I**EGGPIGD**T**LEKISNEI**K**IVATPDGGS
MAL D 1 53 IKKITFGEGSQY**G**YVKH**RI**D**S**ID**EA**SYSYSYTL**I**EGDALTD**T**IEKISYET**K**LVACGS-GS
DAU C 1 53 VRIITLPEGSPI**T**TMTV**RT**D**A**VN**KE**ALSYDSTV**I**DGDILLG**F**IESIETHM**V**VVPTADGGS
PRU AV1 53 IKKITFGEGSQY**G**YVKH**KI**D**S**ID**KE**NYSYSYTL**I**EGDALGD**T**LEKISYET**K**LVASPSGGS
ARA H 8 52 IKKLTIVEDGETKFILHKVESID**EA**NYAYNYSVVGGVALPPTAEKITFETKLVEGPNGGS
API G 1 53 LKIITLPDGGPI**T**TMTL**RI**D**G**VN**KE**ALTFDYSV**I**DGDILLG**FI**ESIENHV**V**LVPTADGGS
GLY M 1 52 IKKITFLEDGET**K**FVLH**KI**E**S**ID**EAN**LGYSYSV**V**GGAALPD**T**AEKITFDS**K**LVAGPNGGS
HYP 1 60 VTKITFVDGHPL**T**YMLH**KF**D**E**ID**AAN**FYCKYTL**F**EGDVLRD**N**IEKVVYEV**K**LEA-VGGGS
FRA A 1 54 IKKITFGEGSHY**G**YVKH**KI**H**S**ID**KVN**HTYSYSL**I**EGDALSE**N**IEKIDYET**K**LVSAPHGGT
consensus 61 ...... ... . ................ .....*... ...*..... ...... .*.

FAG S 1 113 ILKS**T**SKYHTKGDHEIKEDQI**KA**GKEEASGIF**K**AVEAYLLANPAAYH----
BET V 1 113 ILKI**S**NKYHTKGDHEVKAEQV**KA**SKEMGETLL**R**AVESYLLAHSDAYN----
MAL D 1 112 TIKS**I**SHYHTKGNIEIKEEHV**KV**GKEKAHGLF**K**LIESYLKDHPDAYN----
DAU C 1 113 ITKT**T**AIFHTKGDAVVPEENI**KF**ADAQNTALF**K**AIEAYLIAN---------
PRU AV1 113 IIKS**T**SHYHTKGNVEIKEEHV**KA**GKEKASNLF**K**LIETYLKGHPDAYN----
ARA H 8 112 IGKL**T**LKYHTKGDAKPDEEEL**KK**GKAKGEGLF**R**AIEGYVLANPTQY-----
API G 1 113 ICKT**T**AIFHTKGDAVVPEENI**KY**ANEQNTALF**K**ALEAYLIAN---------
GLY M 1 112 AGKL**T**VKYETKGDAEPNQDEL**KT**GKAKADALF**K**AIEAYLLAHPDYN-----
HYP 1 119 KGKI**T**VTYHPKPGCTVNEEEV**KI**GEKKAYEFY**K**QVEEYLAANPEVFA----
FRA A 1 114 IIK**T**TSKYHTKGDVEIKEEHV**KA**GKEKAAHLF**K**LIEGYLKDHPSEYNGSRS
consensus 121 ..*.. ....*.. ..... .* ..... ......*.*... .. .

**Supplementary Figure 1**. Primary sequence alignment of ten proteins from the PR-10 family. Conservation index is mapped by shaded grey scale, magenta boxes indicate residues located at Fab BV16 epitope on Bet v 1 (Mirza et al., 2000) and cyan colored represent conformational epitope of polyclonal antibodies mapped by Asam et al. (1014) (38, 65, 70, 71, 73, 76, 77, 86, 94, 103, 117, 134, 135, 145) (Asam et al., 2014). The epitopes are cyan shaded in both Fag s 1 and Bet v 1 for comparison.


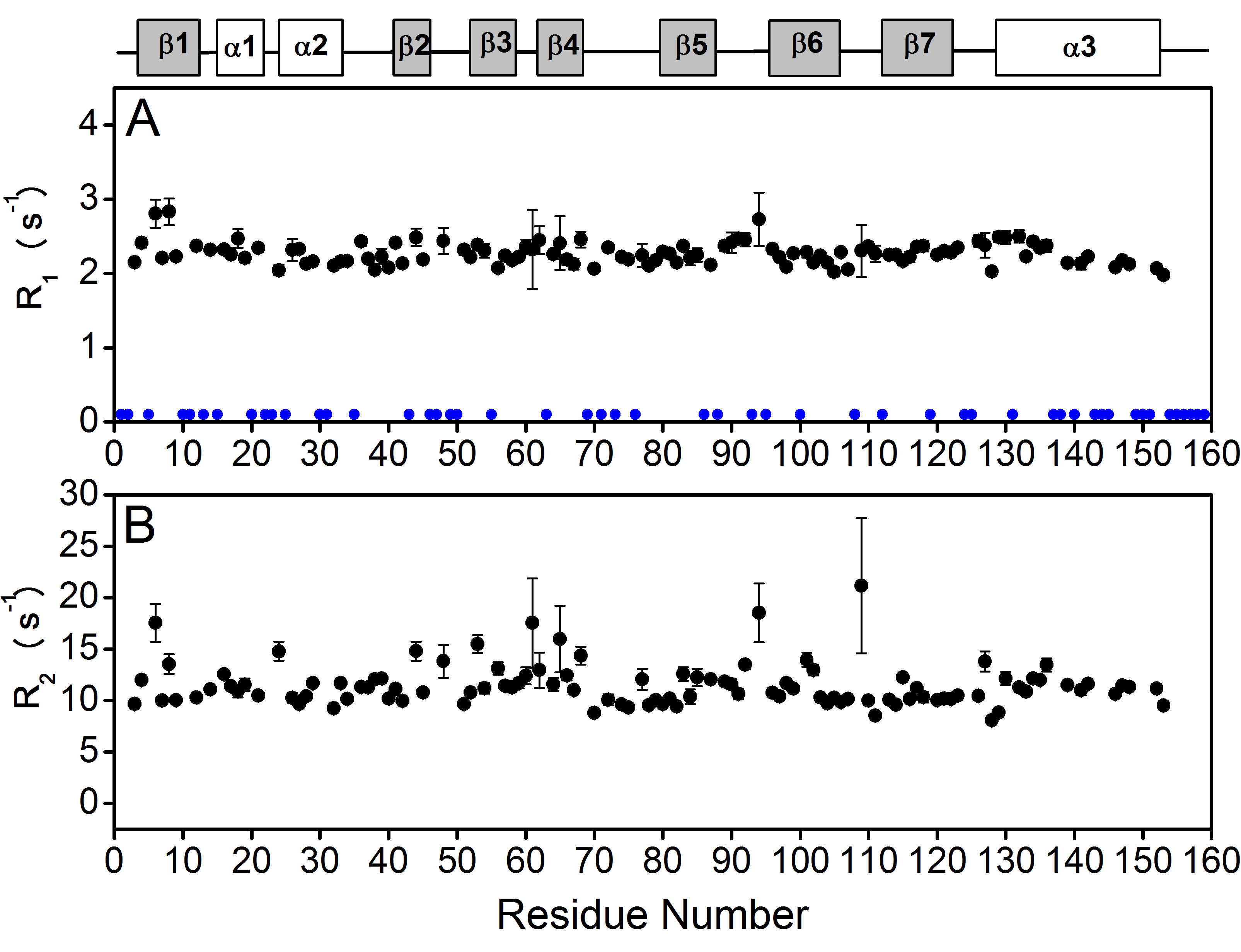


(a)

(b)

**Supplementary Figure 2**. (a)-(b) NMR longitudinal and transversal relaxation rates, R_1_ and R_2_, respectively, as a function of primary sequence of Fag s 1. Experimental rates were obtained from experiments acquired in spectrometer Bruker 500 MHz at 308 K using 200 μM of Fag s 1 diluted at PBS 20 mM, pH 7.8. Blue circles indicate residues for which it was not possible to obtain the relaxation rates due to signal overlap and data with low quality.


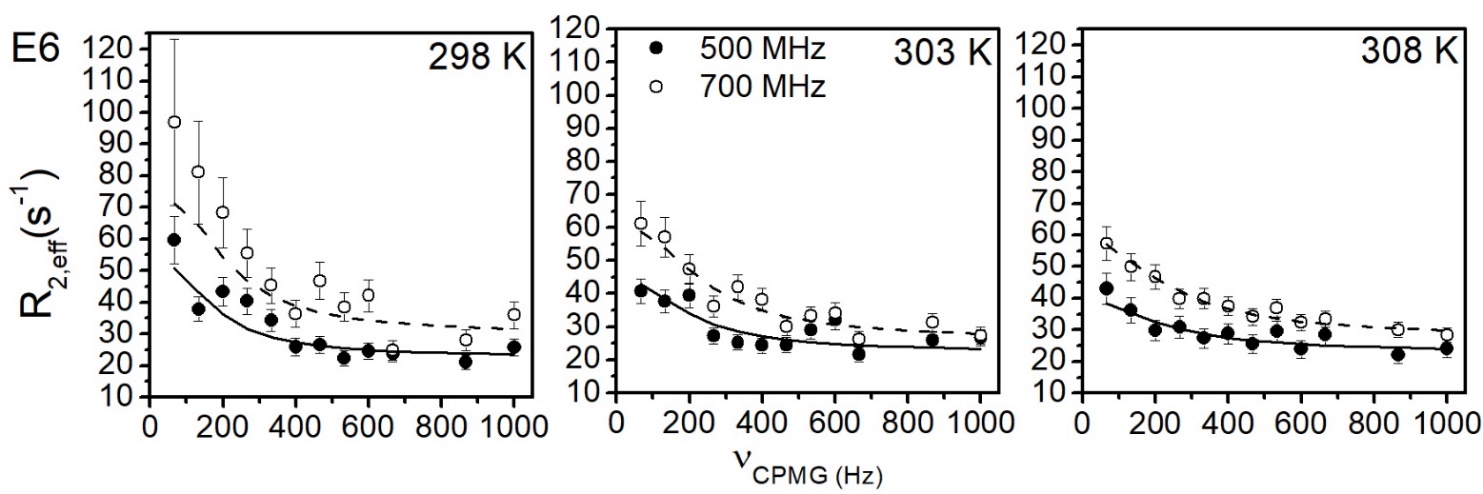

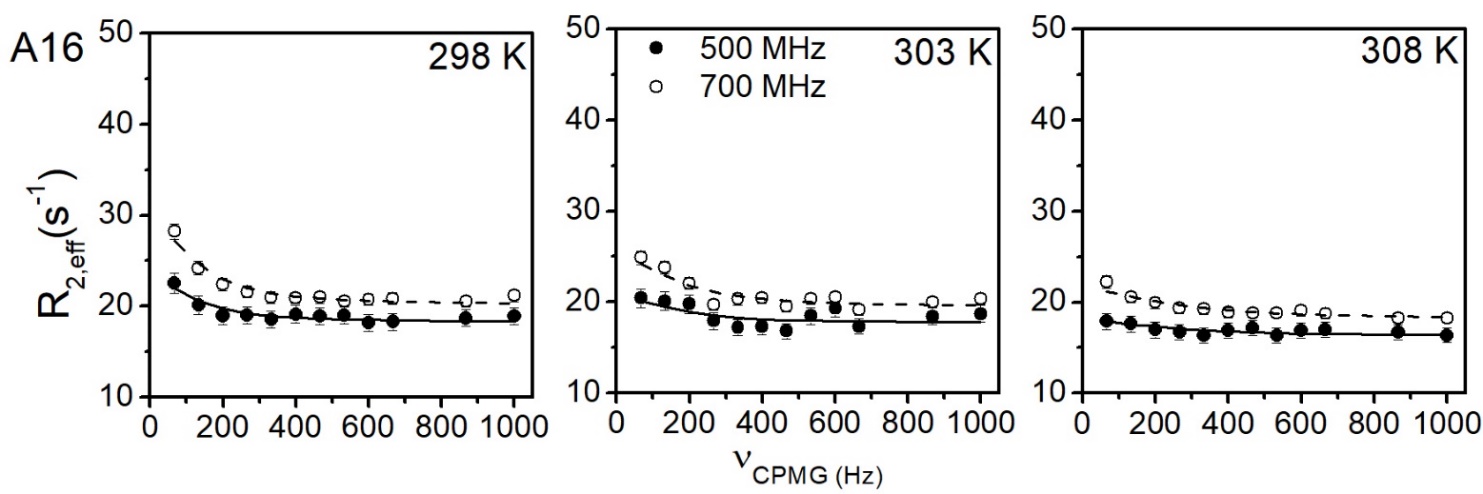

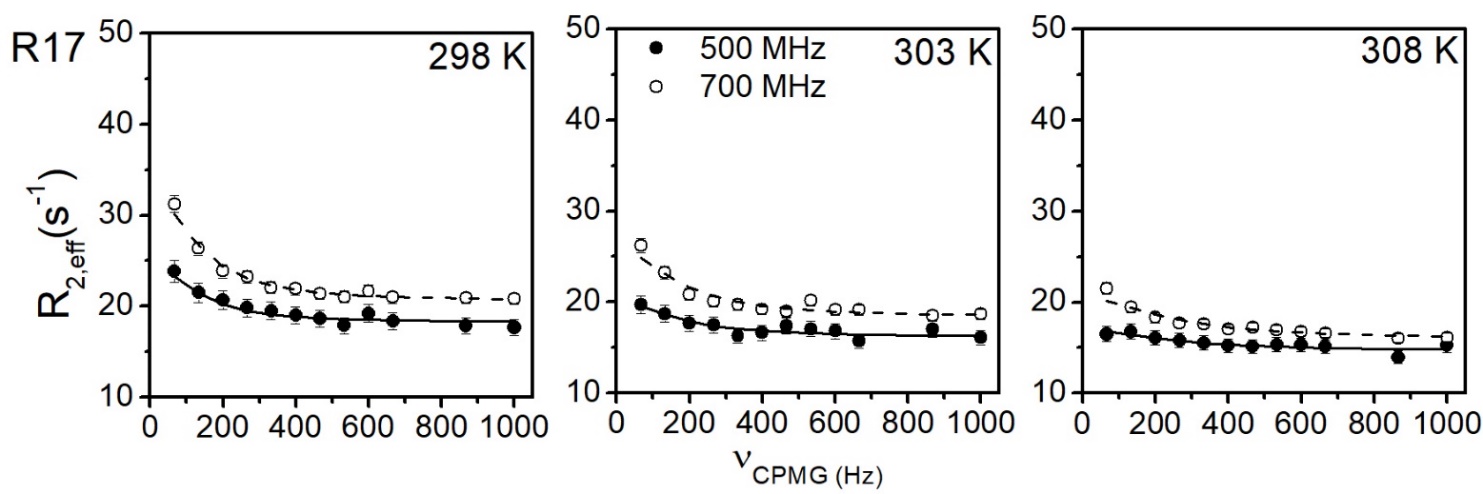

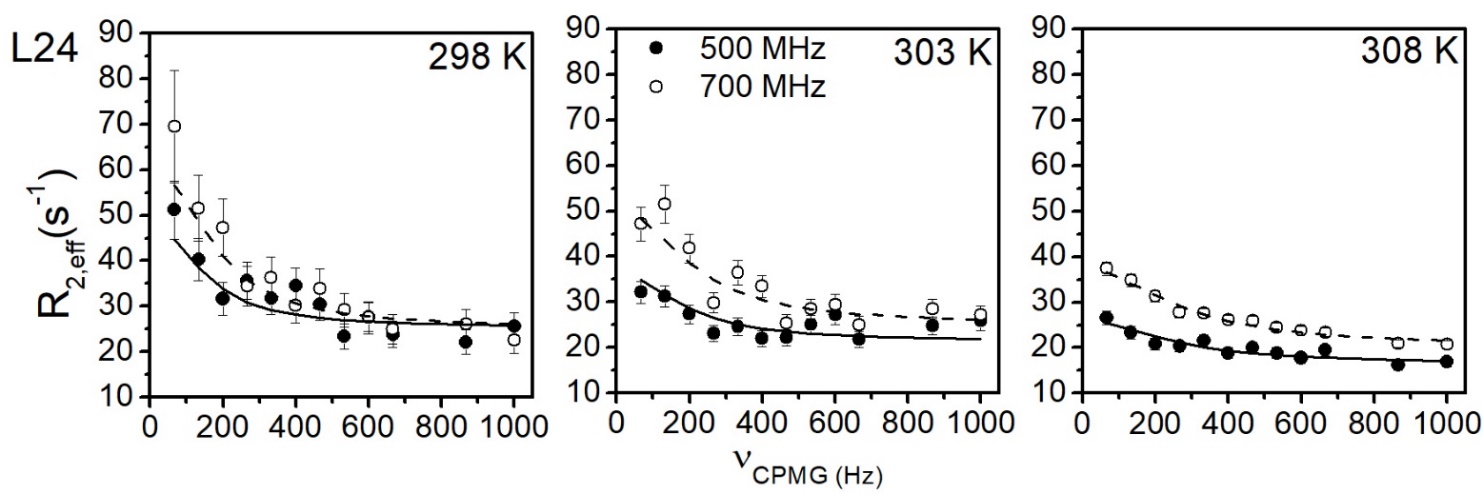

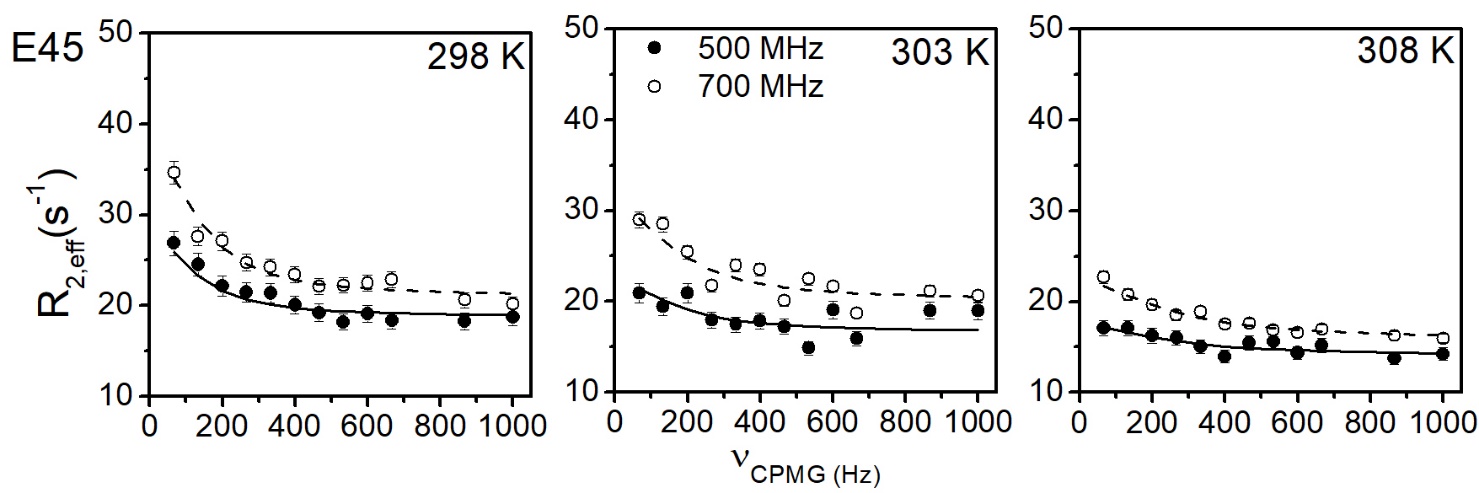

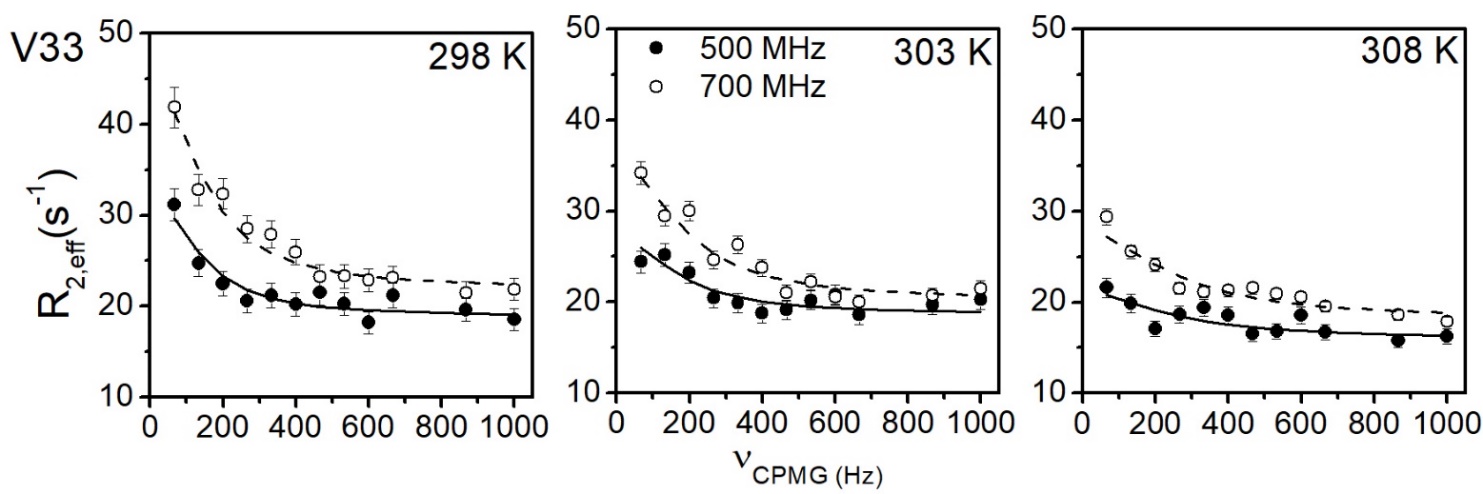

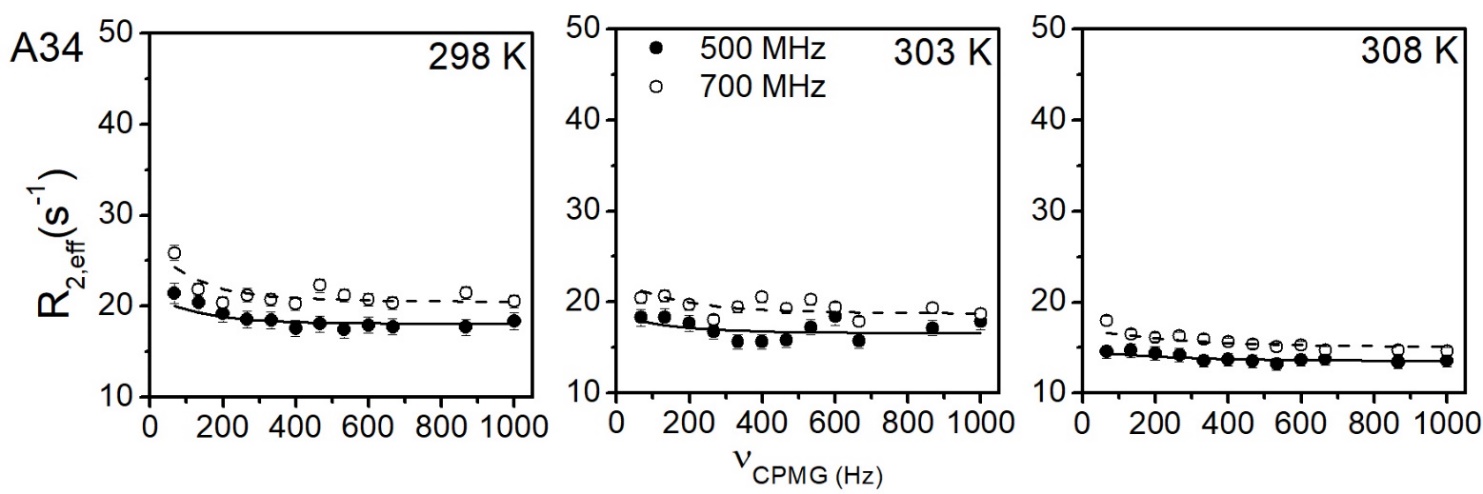

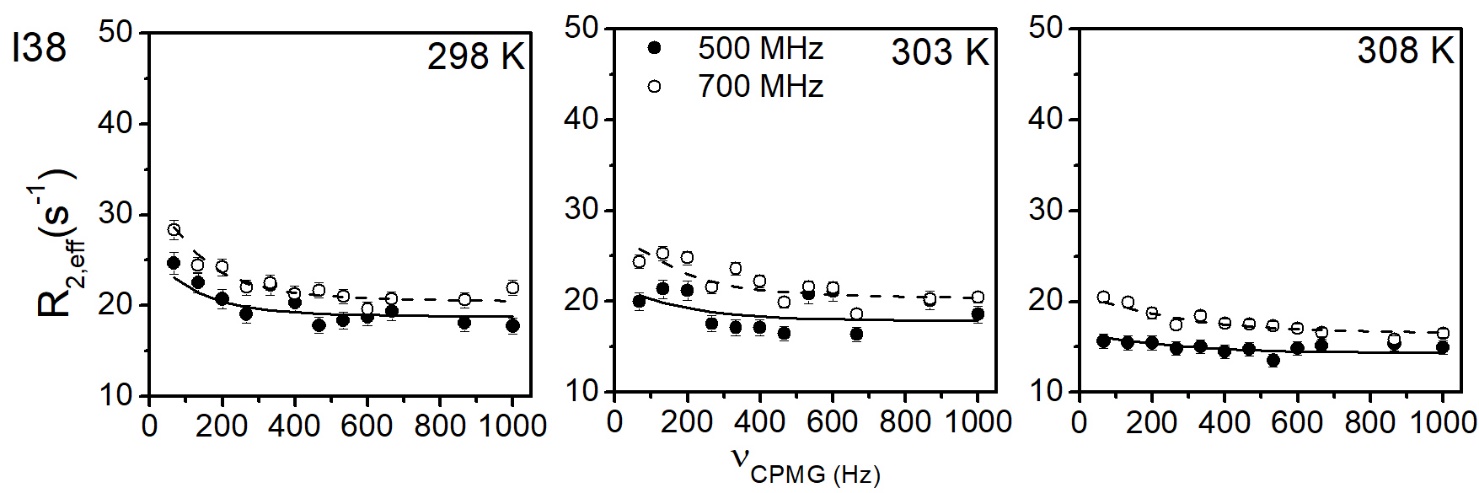

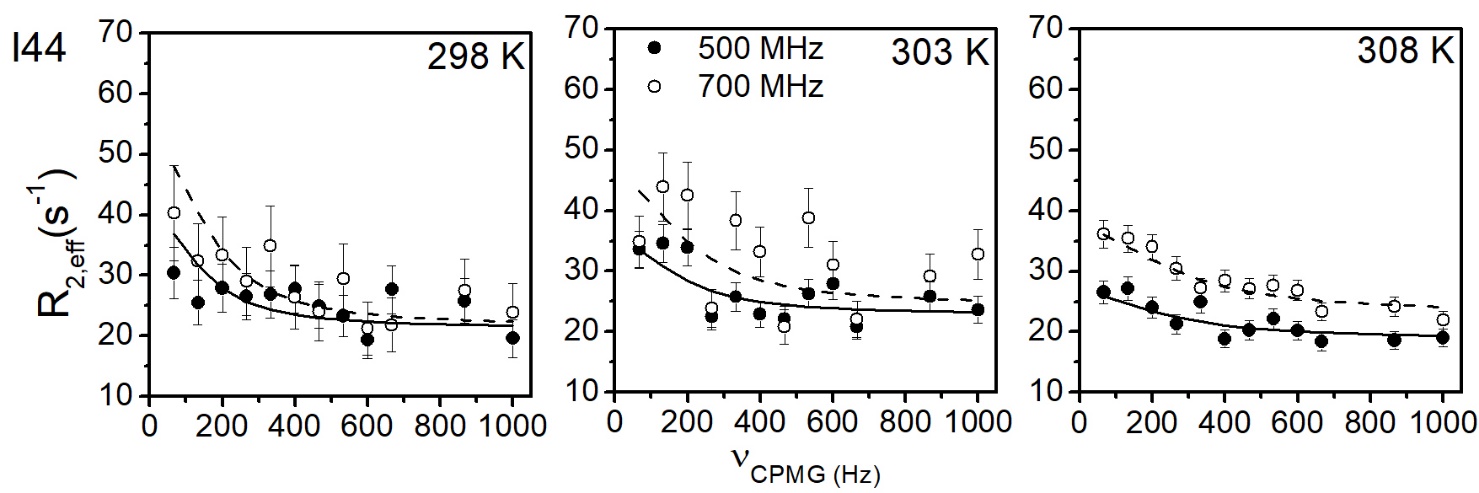

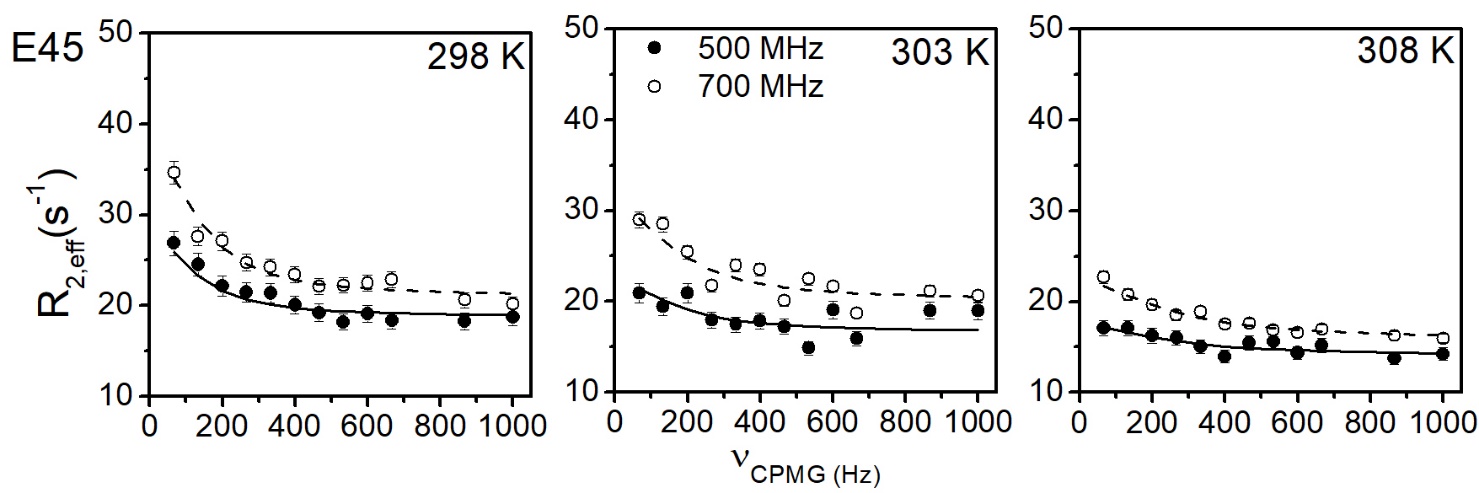

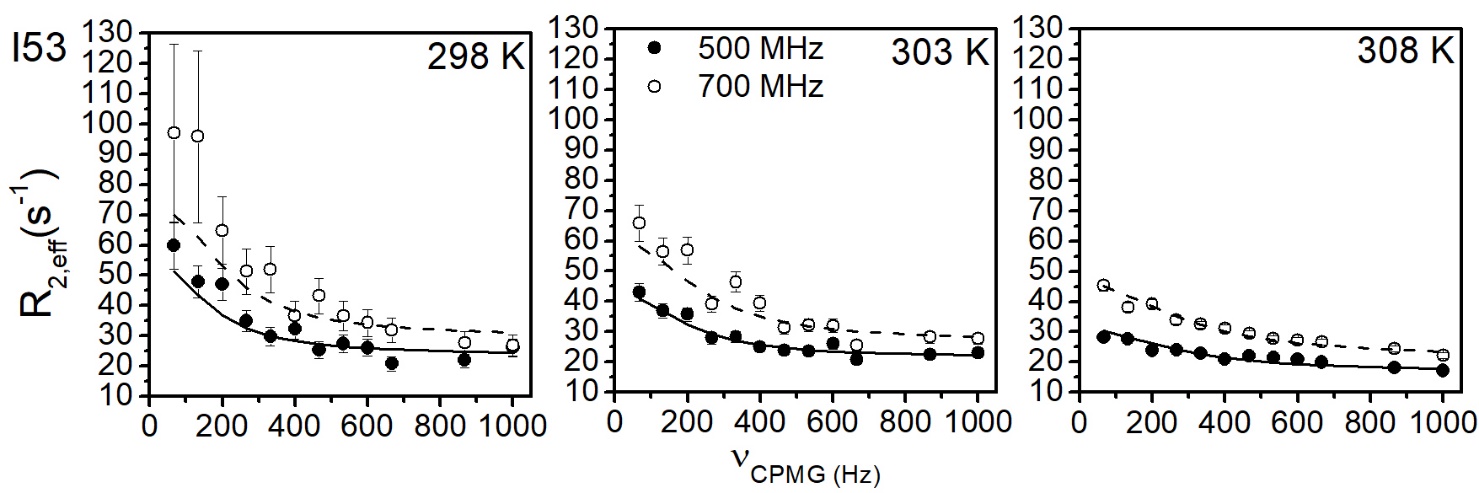

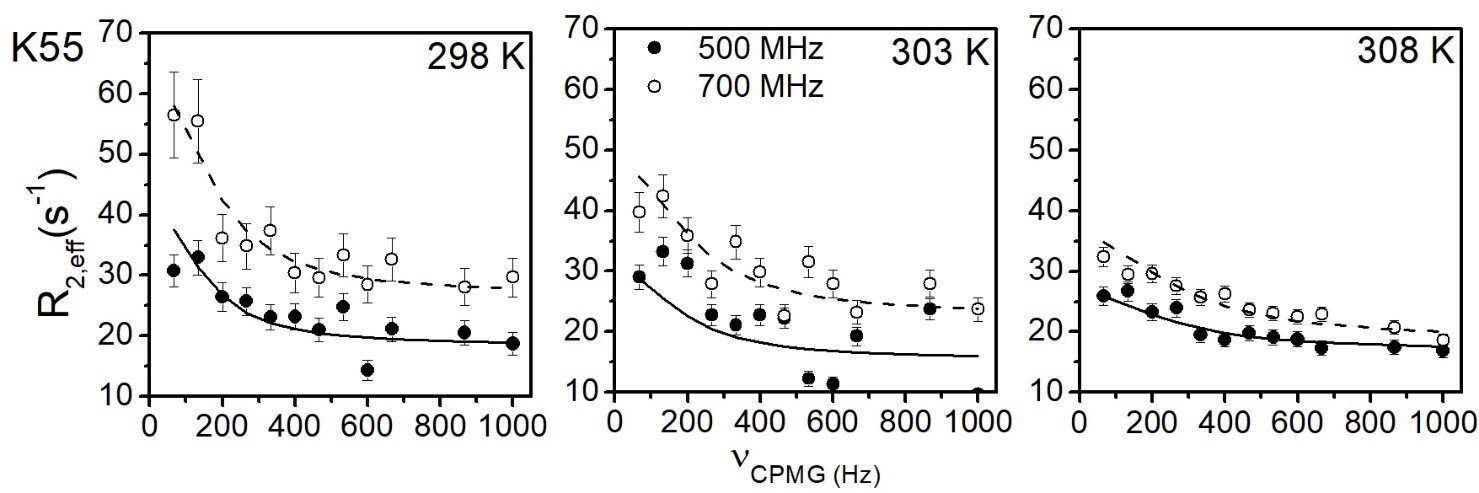

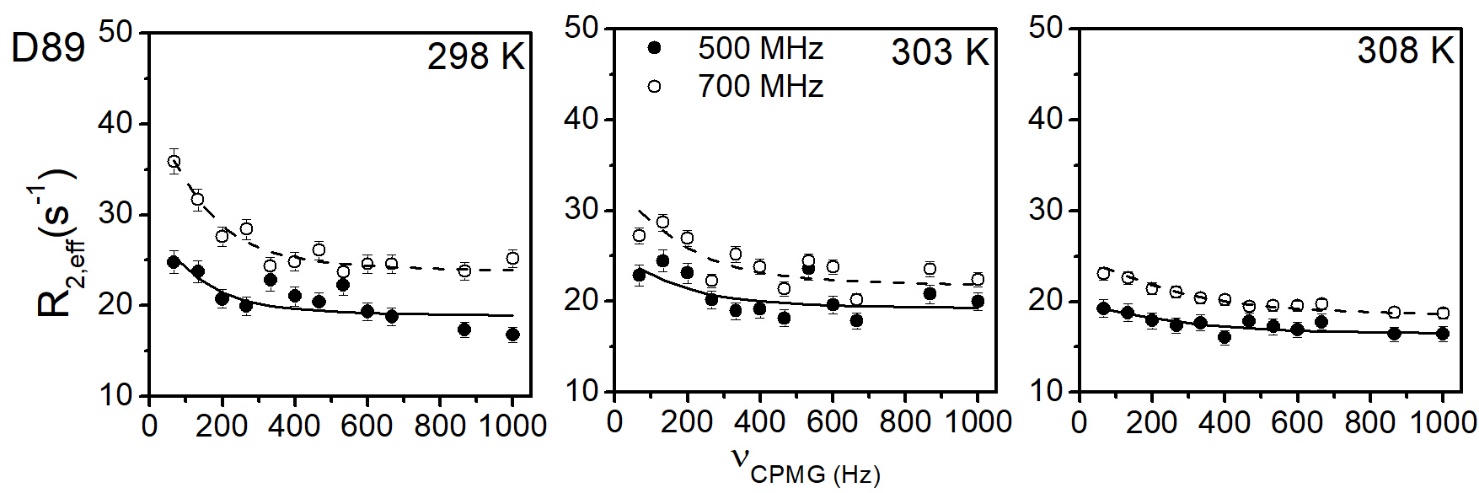

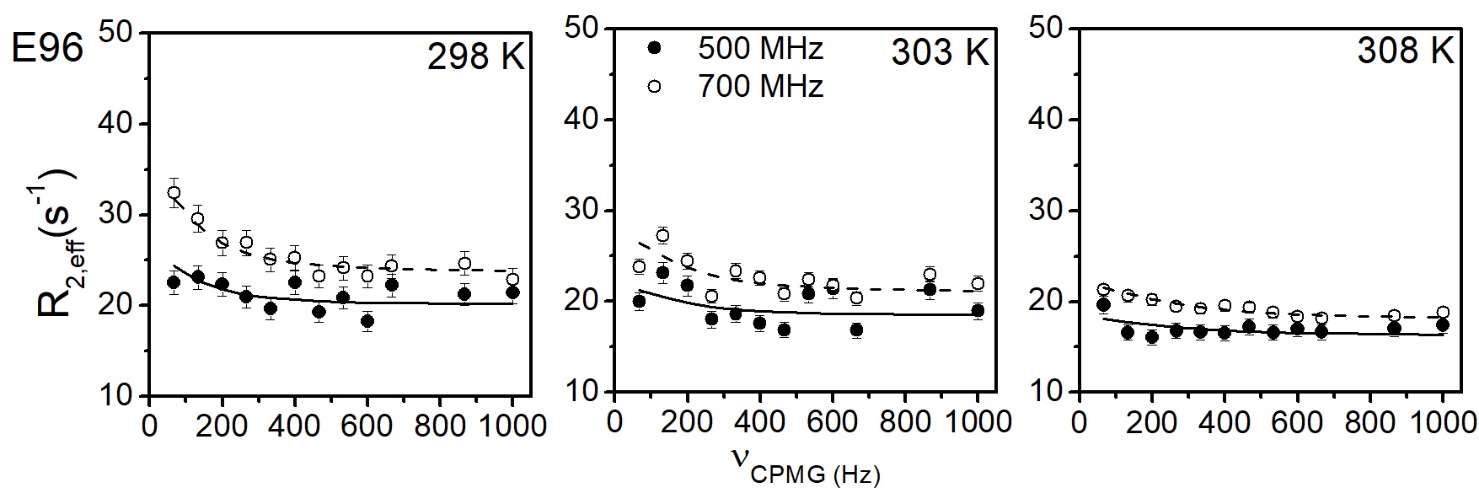

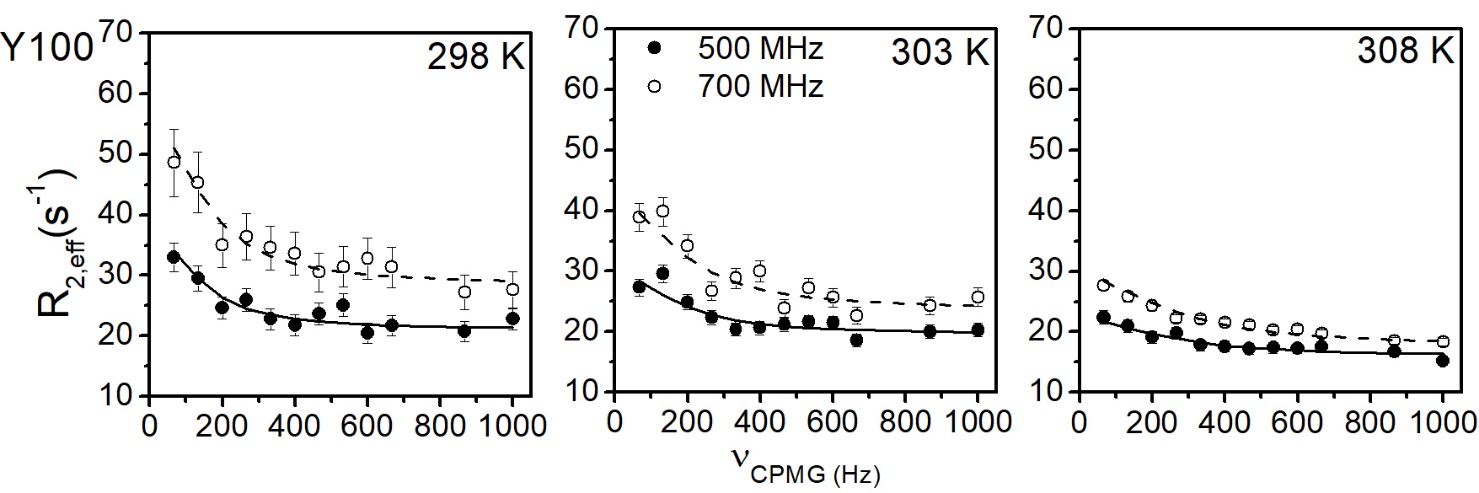

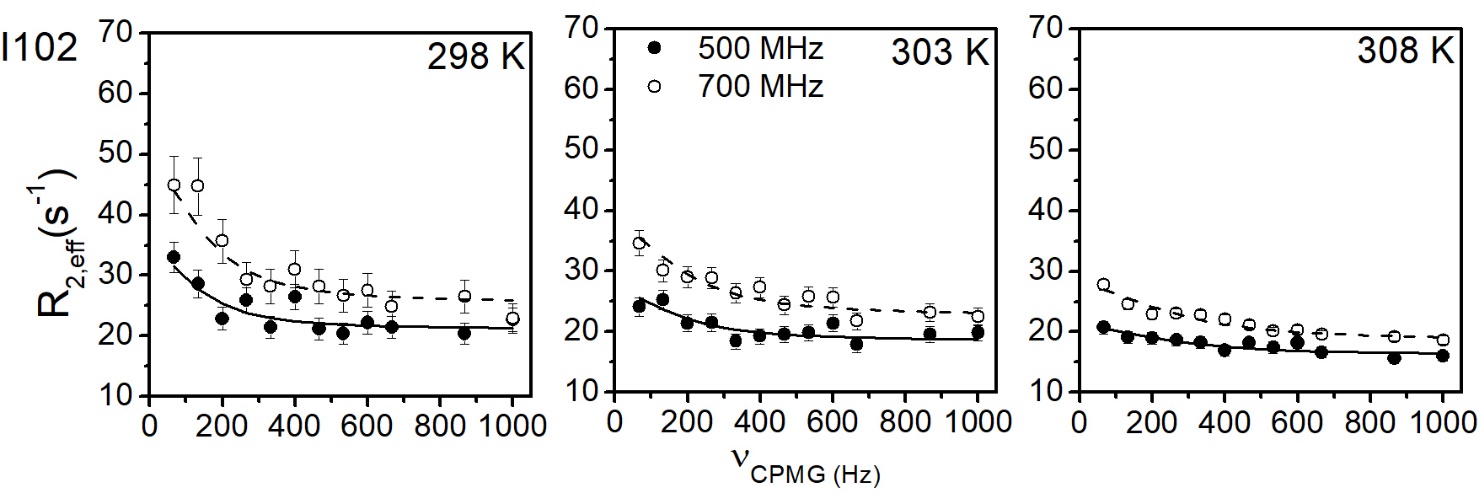

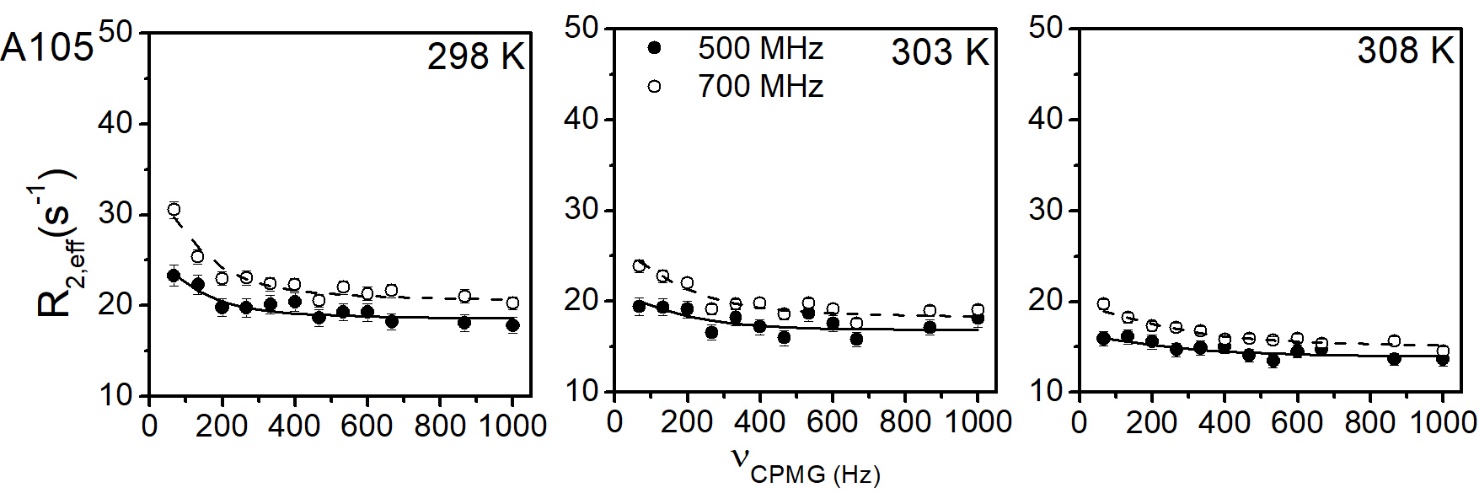


**Supplementary Figure 3:** ^15^N-RC-CPMG relaxation dispersion curves of Fag s 1 residues undergoing conformation exchange and selected as mentioned in Materials and Methods


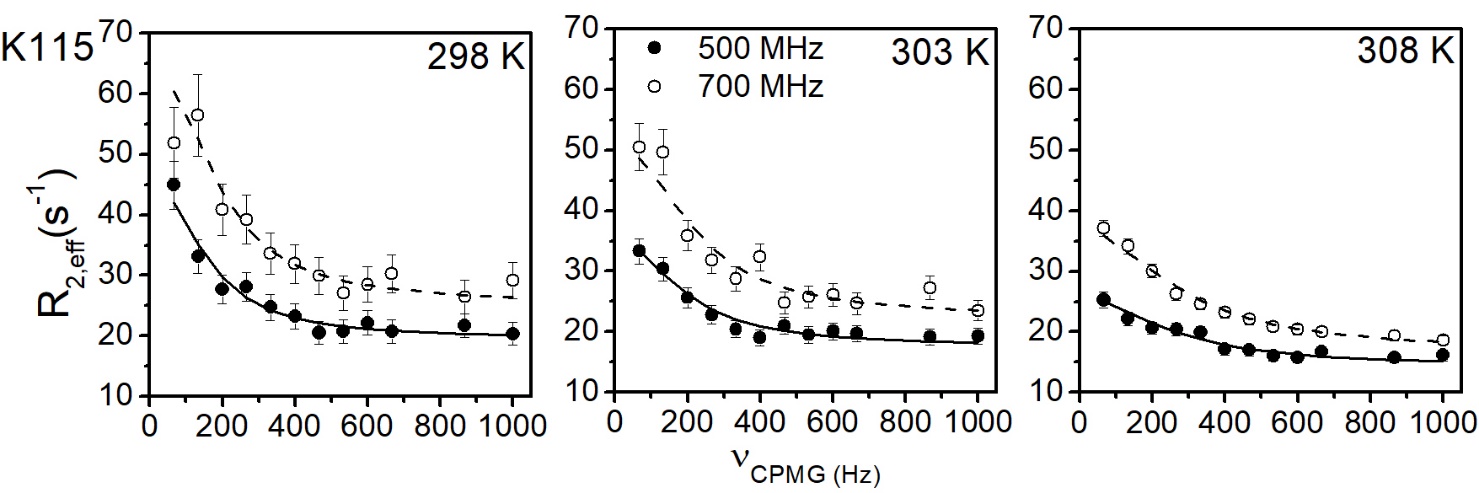

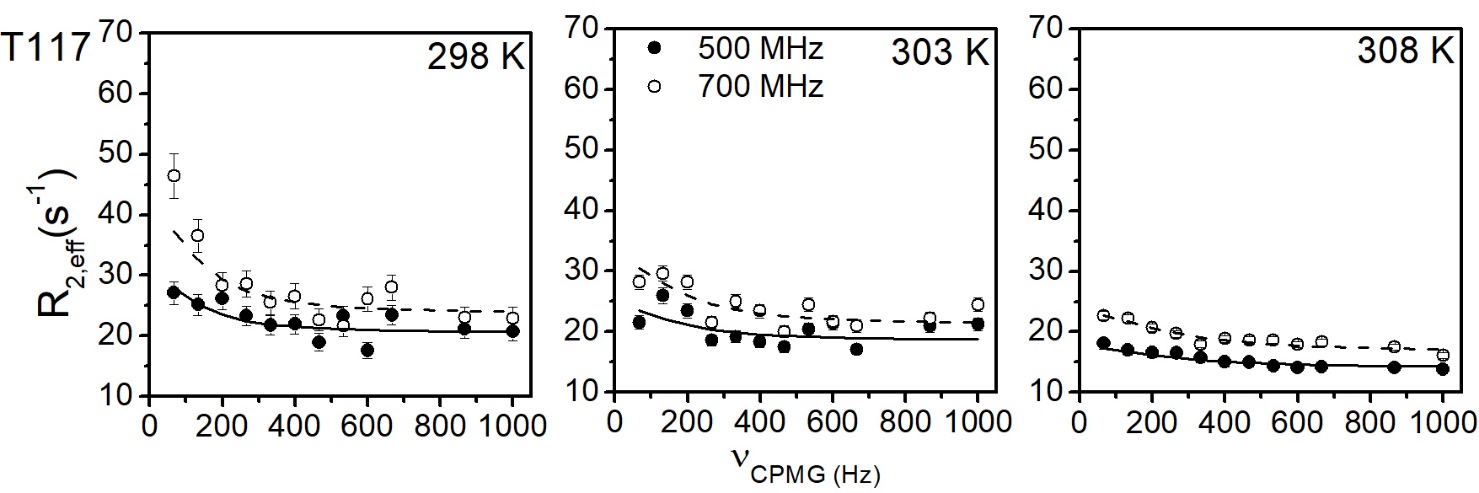

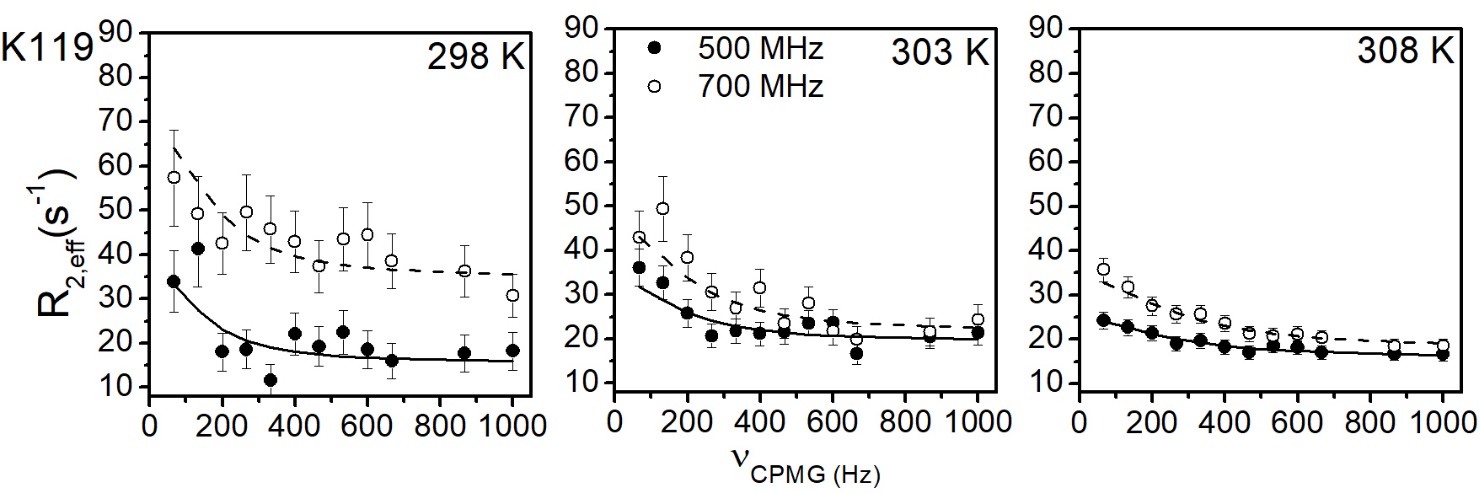

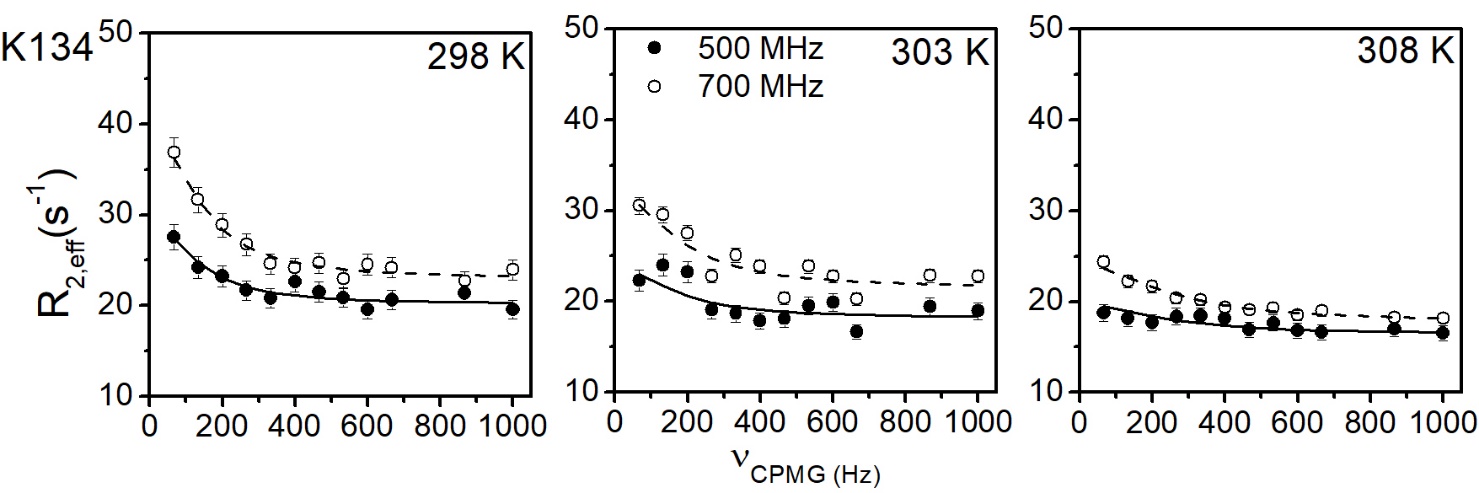

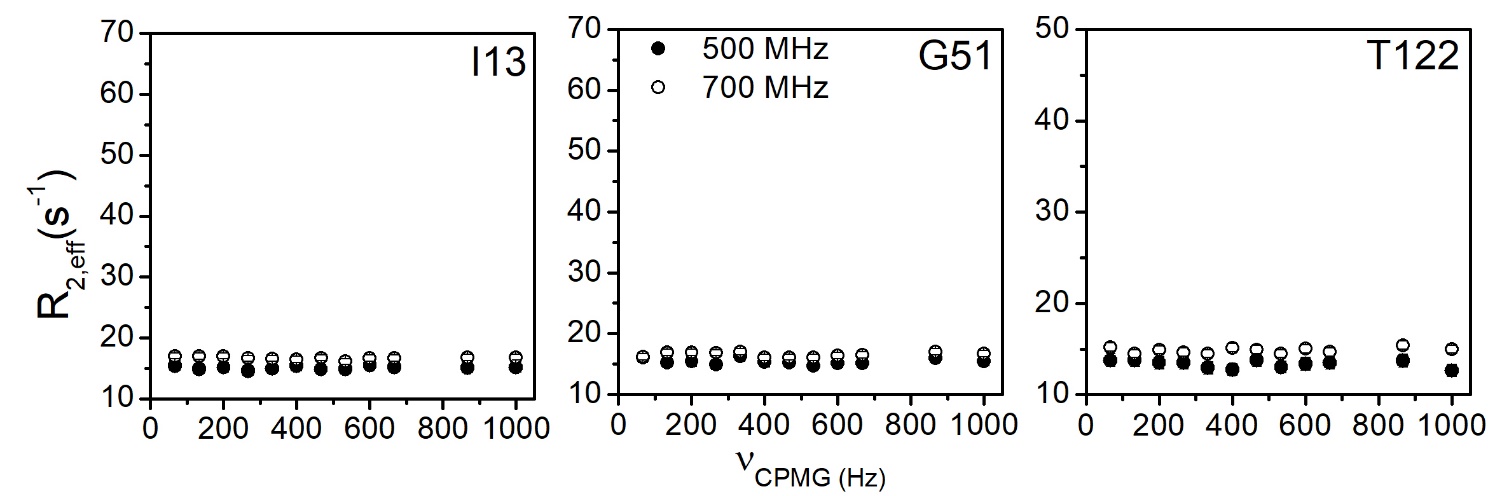

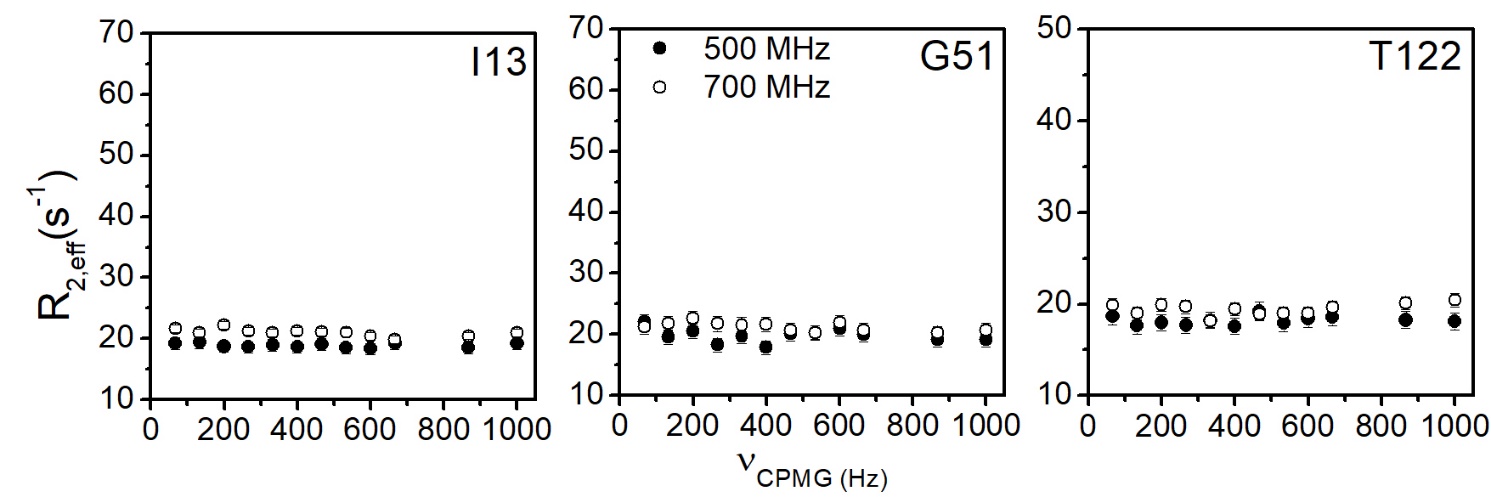


**Supplementary Figure 4:** ^15^N-RC-CPMG relaxation dispersion curves of Fag s 1 residues that are not undergoing conformation exchange at the lowest temperature 298 and 308 K, respectively.


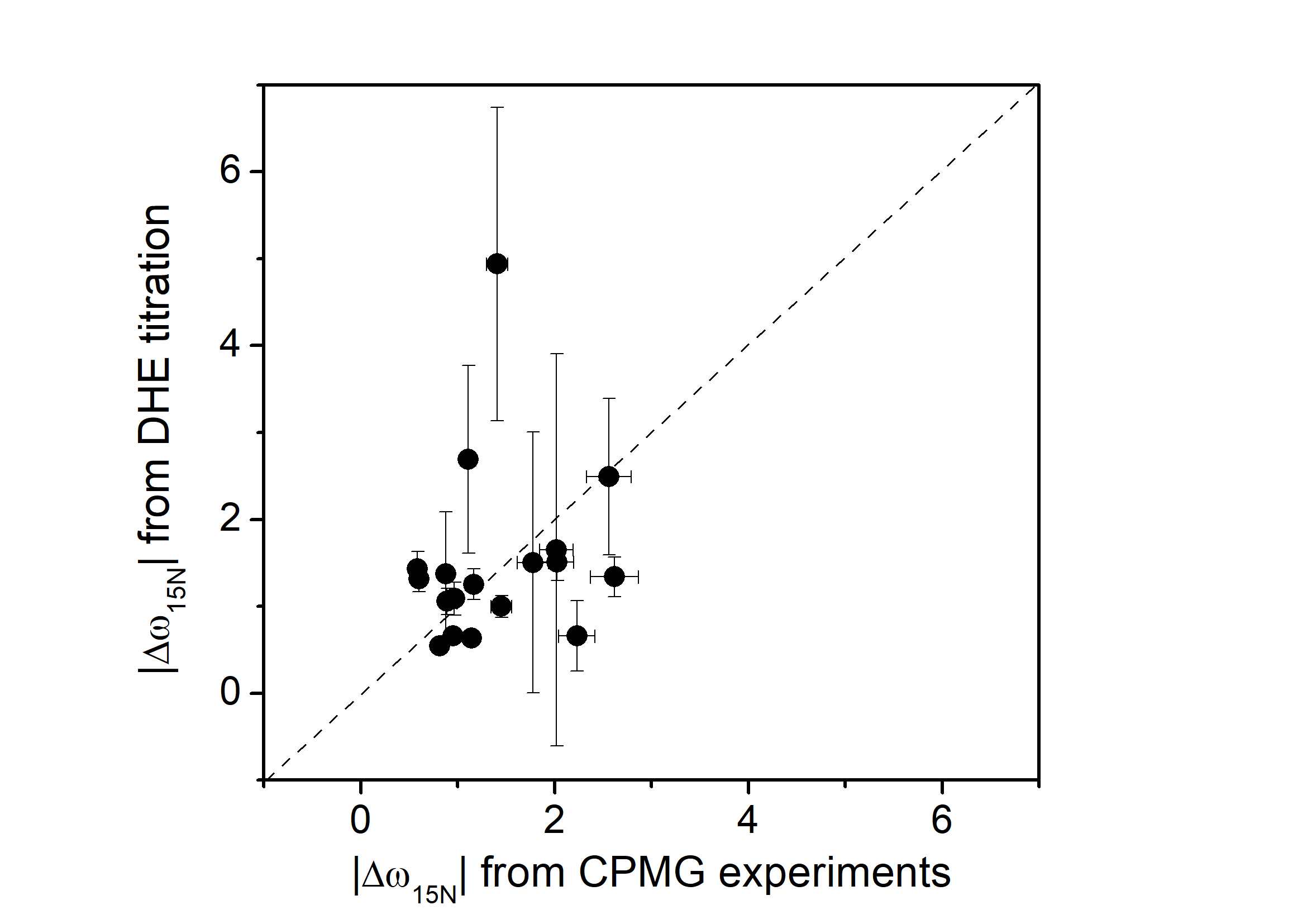


**Supplementary Figure 5:** Correlation Plot of Δω_15N_ information obtained from DHE titration and CPMG experiments.


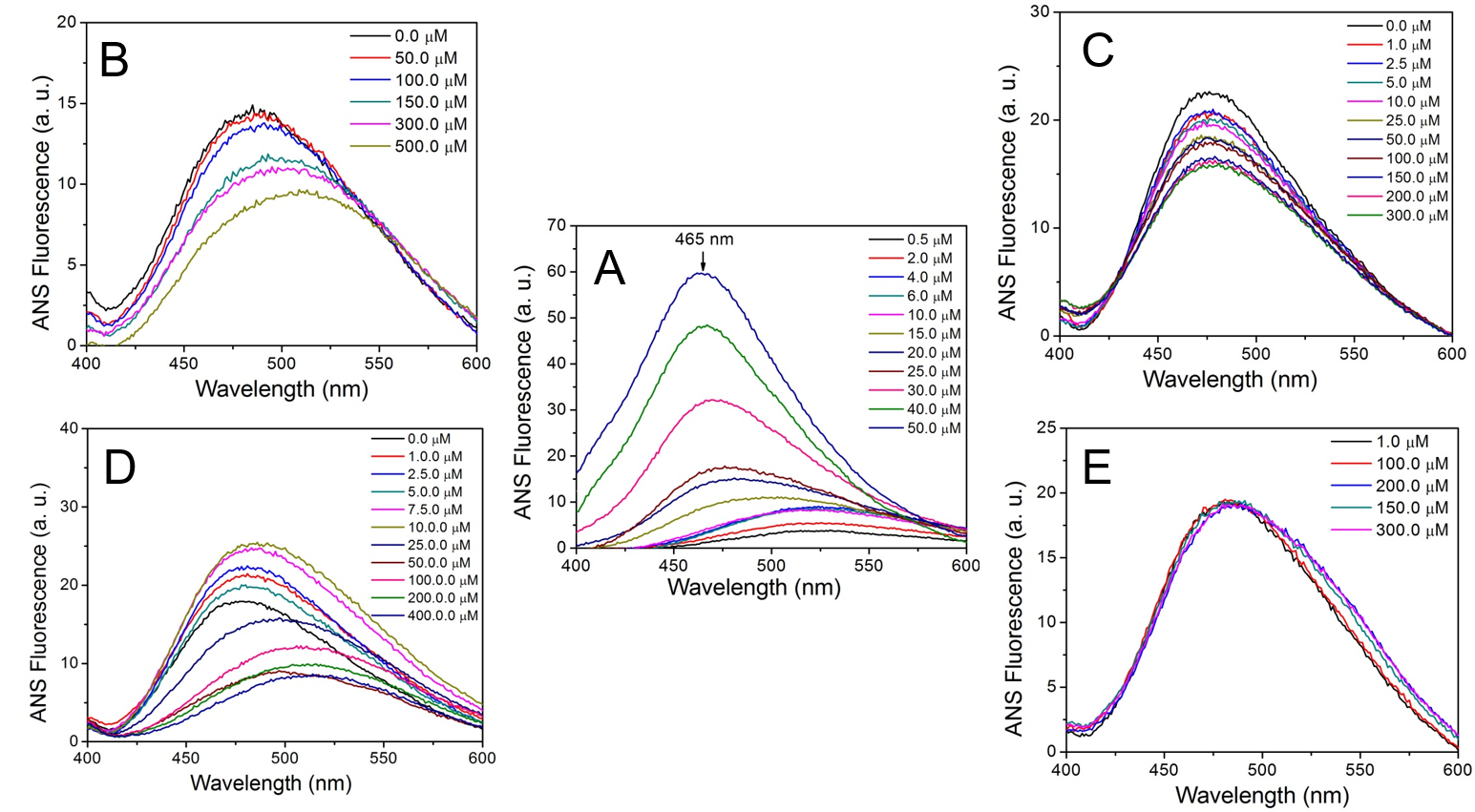


**Supplemental Figure 6.** Titration assay using fluorescence. (a) Fluorescence spectra of 5 μM of ANS with 0.5 to 50 μM of Fag s 1. Competition binding assay of ANS with naringenin (b), kinetin (c), SDS (d) and quercetin (e) using Mogensen et al., 2002 approach. Ligands were titrated in solution with 5 μM of ANS and 15 μM of Fag s 1.

(e)

(d)

(c)

(a)

(b)
